# Vision-Based Artificial Intelligence Technologies for Epilepsy Monitoring: Scoping Review and Taxonomy Development Study

**DOI:** 10.2196/83895

**Published:** 2026-06-24

**Authors:** Mirijana Irnich, Jonas Hammer, Aleksandra Flok, Frank Teuteberg

**Affiliations:** 1 Department of Management Accounting and Information Systems, University Osnabrück, Katharinenstraße 3, Osnabrück, 49074, Germany, 49 5419694926

**Keywords:** epilepsy, taxonomy, artificial intelligence, health care, monitoring, computer vision, classification system, digital health, eHealth, remote patient monitoring systems, health information systems

## Abstract

**Background:**

Artificial intelligence (AI) technologies for vision-based epilepsy monitoring are advancing rapidly in health care. Despite growing research using various video data sources and analytical approaches, no comprehensive framework exists to classify these technologies.

**Objective:**

This scoping review aimed to develop and validate a taxonomy for AI technologies in vision-based epilepsy monitoring and to characterize visual AI approaches in epilepsy care.

**Methods:**

Using an extended taxonomy development framework, we developed the taxonomy in 5 iterative cycles, drawing on theory and practice. We conducted a scoping review, market analysis, and applicability evaluation with market-ready solutions. We searched Scopus, Web of Science, and PubMed, including MeSH (Medical Subject Headings) terms; the final search was completed on January 16, 2026. We included primary studies from 2013 onward on AI-based or machine learning–based monitoring or prediction of epileptic seizures in humans using visual data. We excluded reviews, non-English publications, nonepilepsy studies, studies focused only on electroencephalography or wearables, animal studies, and pre-2013 publications. Evidence was charted through narrative and tabular synthesis and descriptive frequency analysis. In line with scoping review guidance, we did not conduct a meta-analysis or critical appraisal. To assess validity and practical relevance, 9 domain experts evaluated the taxonomy using a Delphi technique.

**Results:**

We included 40 original studies. Study analysis yielded 16 dimensions, including data acquisition source, tracking target, image processing, classifier type, performance metrics, environment, seizure classification, data privacy, and user interface. Expert feedback added 4 further dimensions, including communication mode and information purpose. The final taxonomy comprises 23 dimensions with 102 characteristics. The review identified structural evidence gaps across settings, evaluation maturity, and reporting practices. Detection and classification in stationary settings predominated, whereas predictive approaches and real-time feedback were limited. Deep learning detection methods were common, but performance reporting was inconsistent, and patient-facing functionalities were limited. Privacy safeguards and standardized metrics were often incompletely reported, reducing comparability and maturity assessment. The taxonomy translates these patterns into guidance for benchmarking, procurement evaluation, user interface, and explainable AI design. We synthesized 5 main findings and 10 implications for research and practice. Key challenges concern standardization, seizure prediction, and real-time applicability.

**Conclusions:**

Vision-based AI technologies for epilepsy monitoring are still dominated by proof-of-concept and pilot evaluations, indicating a gap between technical feasibility and deployment-ready systems. This scoping review presents an implementation-oriented taxonomy integrating application context, system architecture, visual analysis, AI models, performance reporting, and feedback design into a single classification framework. Unlike prior work that mainly maps methods or data sources, the taxonomy provides a shared structure for consistent system-level characterization and comparison across studies and emerging solutions. It may support benchmarking, implementation-focused evaluation, procurement, and translation into clinical and home settings.

## Introduction

### Background

Epilepsy, a prevalent neurological disorder affecting approximately 50 million individuals globally, is characterized by recurrent, unprovoked seizures that can significantly impair quality of life [1,2]. Effective monitoring and timely detection of seizures are crucial for optimal management and improved patient outcomes, especially for preventing sudden unexpected death in epilepsy [3]. Traditional monitoring methods, such as electroencephalography (EEG), often require wearable devices that can be intrusive and uncomfortable, potentially limiting patient compliance and continuous monitoring capabilities [4]. Recent advancements in artificial intelligence (AI) have paved the way for innovative, nonwearable monitoring systems that use audiovisual data to predict, detect, and warn of epileptic seizures [5]. These systems offer a contactless approach, enhancing patient comfort and enabling continuous monitoring without the physical constraints associated with wearable devices [6]. For instance, vision-based AI systems have demonstrated efficacy in detecting motor seizures by analyzing patient movements in visual data, most commonly video recordings, providing a nonintrusive alternative to traditional methods [7].

The current state of the art in epilepsy monitoring remains largely centered on EEG due to its high accuracy in detecting seizure events [8,9]. EEG-based systems remain the clinical gold standard, offering precise detection capabilities, but they are often resource-intensive because of long setup times, the need for clinical expertise, and the associated long hospital stays [3]. This traditional approach relies heavily on inpatient settings, wearable devices, or induced seizures through medication withdrawal—methods that can be intrusive, resource-intensive, and distressing for patients [10-12]. Given the high costs of hospital stays and long waiting times for diagnostic assessments, home telemonitoring has gained increasing importance [11]. Consequently, there is an emerging demand for innovative, patient-friendly monitoring systems that address these patient needs. For individuals with epilepsy, this involves reducing dependence on hospital-based diagnostic procedures through the implementation of reliable home-based detection systems. This approach is expected to ensure timely interventions, thereby enhancing the general well-being and quality of life of those affected.

A promising development in the field has been the combination of EEG with video-based seizure detection, which integrates the accuracy of EEG with the nonintrusive nature of vision-based monitoring [12]. Advances in deep learning (DL) have further enhanced vision-based seizure detection, improving system performance and providing additional decision support for epilepsy management [13]. These improvements have facilitated a transition from clinical technologies to home-based solutions relying solely on video monitoring, enabling greater accessibility and continuity of care [8]. The ability to capture seizure events in real-world settings further enhances ecological validity and contributes to more personalized treatment approaches [13]. The integration of AI with video data not only alleviates the discomfort associated with wearable devices but also enhances the accuracy of seizure detection. AI algorithms can analyze subtle visual cues and patterns that indicate seizure activity, facilitating early detection and intervention [4,5]. Moreover, nonwearable systems reduce the burden on patients and caregivers, promoting better adherence to monitoring protocols and improving overall quality of life [3].

AI monitoring systems represent a transformative shift in epilepsy detection and management, with vision-based solutions emerging as a key advancement in the field. These systems follow a similar workflow to process video data and analyze it using AI techniques. An illustrative overview of an exemplary schematic flow of monitoring systems based on visual data with AI models is provided in Multimedia Appendix 1. To facilitate a comprehensive classification of the contents of this work, the 5 steps of the process that serve as a contextual foundation are presented below as examples: monitoring, visual data acquisition, visual data preprocessing, AI model processing, and event analysis. At the start of the schematic flow, patient monitoring is initiated using either mobile or stationary systems to enable continuous observation [3,14]. Visual data are acquired through optical sensors that generate video recordings, which are subsequently preprocessed. The goal of preprocessing is to reduce artifacts, that is, unwanted disturbances or distortions that alter the signal, may compromise analytical quality, and are not part of the original input [15]. Common methods include image denoising, video stabilization, and normalization of video data to ensure high-quality input for subsequent AI-based analysis [14]. AI model processing then uses these optimized data, often through DL or convolutional neural networks (CNNs), to identify patterns and estimate seizure probabilities. Preprocessed data are analyzed with neural networks to detect potential clinical events, such as tonic-clonic seizures. Event analysis involves validating these potential events against predefined thresholds and classification criteria. If thresholds are exceeded (eg, event duration or intensity), the events are systematically classified as clinically relevant [16,17]. Automatically generated reports provide detailed information, including the temporal characteristics of the detected events [18]. In addition, a review process may be conducted in which medical professionals validate automated classifications and diagnoses and, when necessary, correct identified events to enhance the accuracy of future AI evaluations [5,18]. Depending on the objective, these monitoring systems either serve seizure detection or support diagnostic processes related to epileptic seizures [5].

The range of applications extends from contactless monitoring of respiratory and heart rates via ultra-wideband or millimeter-wave radar to continuous sleep analysis, reconstruction of mechanical and electrical cardiac activities, and analysis of nocturnal breathing patterns to detect early signs of neurodegenerative diseases, such as Parkinson disease [19]. Moreover, movement sequences are recognized and classified, which is particularly relevant for automatic fall detection in older or care-dependent individuals [20]. Another application includes monitoring correct medication intake, enabling the early identification of potential errors in administration [19]. As vision-based technologies advance, they are anticipated to serve as a fundamental component of future epilepsy monitoring, providing a less invasive and more accessible alternative to traditional methods. While challenges remain, such as ensuring system reliability, addressing environmental variability (eg, fluctuating light conditions or disturbances), and improving user training, ongoing advancements in AI and DL are rapidly enhancing system performance [5,11,13,21,22]. With continuous technological progress, vision-based AI systems have the potential to transform epilepsy care by providing accessible, cost-effective, and user-friendly monitoring in real-world environments.

Previous reviews in this domain have provided valuable but largely isolated insights into specific aspects of AI-based epilepsy monitoring. Their categorizations primarily focused on the scope of monitoring applications [13], target groups such as pediatric or adult patients [3], the period of epilepsy (eg, ictal vs interictal) [3,10], data acquisition sources including EEG and video [23], tracking targets [13], video tracking approaches [21], image processing methods [24,25], and the types of classifiers and performance metrics used [23]. While these contributions have mapped important components of the field, they remain fragmented and mainly descriptive. Whereas earlier reviews have summarized the evidence, our work formalizes it by developing a taxonomy that specifies mutually exclusive and collectively exhaustive dimensions and characteristics. Their initiation occurs on the premise of substantiated proof-of-concept and concrete systems, with refinement occurring through iterative design methodologies. Our taxonomy integrates technical, clinical, and contextual dimensions into a single, coherent schema that enables consistent classification across research and market solutions of AI-enabled video monitoring for epilepsy. By synthesizing these disparate elements into a unified conceptual framework, the taxonomy provides a structured foundation for systematically comparing existing systems, identifying research gaps, and guiding the design and evaluation of future AI-driven monitoring solutions.

### Objectives

Current research leverages diverse data sources and technological methods to advance the field of vision-based AI monitoring [8-10]. Despite prior studies conducting reviews to provide overviews of these approaches [11-14], a structured taxonomy for categorizing these developments remains absent, and little is known about the dimensions and characteristics of AI technologies in vision-based epilepsy monitoring. To address this research gap, this scoping review developed a taxonomy following the method proposed by Nickerson et al [26], which prescribes iterative cycles to identify dimensions and characteristics, and systematically examined AI technologies in vision-based epilepsy monitoring in both research and market technologies. Specifically, this paper clarified the purpose of developing a taxonomy by articulating why it is needed, how it is constructed, and what it contributes based on the extended taxonomy design process (ETDP) [16]. By reviewing the literature and the health care market and incorporating expert insights, it provides a structured overview of existing solutions and their state-of-the-art developments and offers a foundation for future advancements.

This scoping review aimed to develop and validate a comprehensive taxonomy for vision-based AI technologies in epilepsy monitoring. Specifically, we derived core dimensions and defining characteristics from a scoping review and a market analysis, instantiated and refined the taxonomy through iterative design cycles guided by an ETDP, and evaluated its completeness and practical relevance using expert elicitation. In addressing research question (RQ) 1, the taxonomy’s dimensions and characteristics are specified. In addition, RQ2 synthesizes implications and future directions for research and practice. Our final taxonomy serves as a structured framework for classifying AI technologies in vision-based epilepsy monitoring, supporting researchers in organizing the field, developers in creating targeted solutions, and decision-makers in evaluating and selecting appropriate technologies. Furthermore, this taxonomy supports businesses and health care institutions in assessing market-ready systems and regulatory compliance while facilitating a systematic understanding of emerging trends for policymakers.

The following RQs will be addressed:

- RQ1: *What are the dimensions and characteristics of AI technologies in vision-based epilepsy monitoring that can be integrated to develop a comprehensive taxonomy?*
- RQ2: *What implications and future directions can be identified from the existing literature and expert perspectives on AI technologies in vision-based epilepsy monitoring?*

## Methods

### Overview of Taxonomy Design Process

A taxonomy is built through iterative derivation, instantiation, and evaluation to define the field’s structure, yielding coherent dimensions and characteristics for comparison and decision-making. This work followed an integrated single evidence base with dual outputs design. A scoping review was conducted to provide the empirical basis for both (1) evidence charting and gap mapping and (2) taxonomy development. These complementary outputs pursue a shared objective of structuring and interpreting a heterogeneous, rapidly evolving body of literature at different analytical levels. This design avoids duplication while ensuring methodological coherence between evidence synthesis and framework construction. The scoping review synthesizes the breadth of published work and enables transparent reporting of study characteristics and evidence gaps. Building on this empirical synthesis, the taxonomy translates the identified patterns into a structured, reproducible classification framework that supports consistent comparison across research prototypes and market-ready solutions.

The aim of this scoping review was to systematically classify emerging AI technologies in vision-based epilepsy monitoring through the development of a comprehensive taxonomy. This was accomplished by adopting the methodological framework [26] and incorporating the ETDP [27]. The ETDP facilitates the explicit description of the underlying problem, the observed phenomenon, the target user groups, and the intended purpose. It is organized along the 6 Design Science Research activities—problem identification, objective definition, design and development, demonstration, evaluation, and communication—and introduces iterative entry and exit points to structure cycles of conceptual and empirical work. For evaluation, ETDP adopts the *why-how-what* framing: clarify the purpose (formative or summative), select the paradigm and techniques (artificial or naturalistic), and specify the evaluated properties, for example, usefulness (Multimedia Appendix 2). *Why* clarifies function, whether the evaluation is formative or summative [28]. *How* specifies the environment, if it is an artificial (eg, laboratory studies) or naturalistic evaluation (eg, case studies with real users) and the timing (*ex-ante* or *ex-post*) and the methods [28,29]. *What* focuses on the criteria, including the objective and subjective ending conditions and the defined evaluation criteria [29]. Overall, the integration of ETDP enables rigorous ex-ante and ex-post assessment of taxonomies while maintaining flexibility for evolving phenomena.

**Figure 1.**
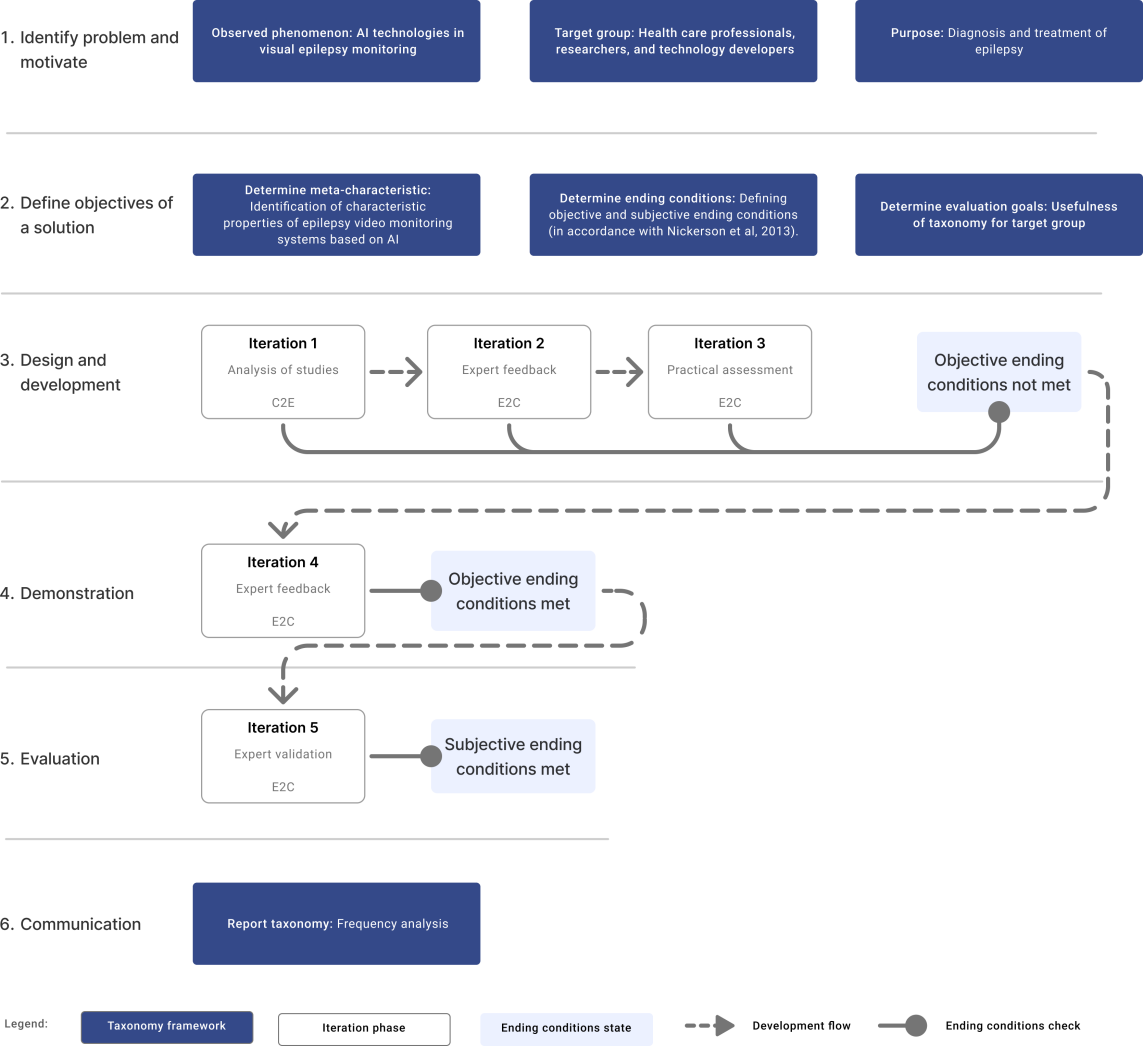
Methodological approach for taxonomy development modeled on Nickerson et al [26]. AI: artificial intelligence.

The iterative taxonomy development process followed either a conceptual-to-empirical (C2E) or an empirical-to-conceptual (E2C) approach [26]. The deductive C2E method initiated the taxonomy development by systematically analyzing the relevant literature in the field of emerging AI technologies in vision-based epilepsy monitoring. An inductive E2C strategy was used to derive dimensions and characteristics from existing solutions in practice. Multimedia Appendix 3 illustrates the ETDP-aligned process and the 5 iterative cycles conducted. The meta-characteristic of this taxonomy is thus the identification of the defining properties of AI technologies in vision-based epilepsy monitoring. The ending conditions were guided by the objective and subjective criteria [26] (Multimedia Appendix 2). To derive the final taxonomy, a structured 5-iteration development process was conducted, alternating between C2E and E2C approaches. This iterative procedure combined theoretical grounding with practical assessment, with the latter achieved through an analysis of market-ready solutions to ensure relevance to current practice and to confirm practical alignment of this taxonomy (Figure 1).

This methodological approach is particularly suited, given the increasing complexity and heterogeneity of AI- and vision-based monitoring solutions in epilepsy care, which currently lack a unified framework to systematically organize and compare their diverse characteristics. Accordingly, the taxonomy development began with a rigorous articulation of the motivation and necessity for such a structured classification within this domain. The observed phenomenon comprises emerging AI technologies in vision-based epilepsy monitoring, especially those using video data such as video-EEG or stand-alone video-based seizure detection systems, which are playing an increasingly pivotal role in the diagnosis and management of epilepsy. The primary purpose of the taxonomy is to enable a systematic and transparent categorization of these emerging AI technologies, thereby fostering conceptual clarity, facilitating informed clinical and technological decision-making, and uncovering relevant avenues for future research. The taxonomy thus aims to address the needs of health care professionals, researchers, and technology developers by providing a comprehensive overview of existing and emerging solutions. Patients and patient advocacy groups represent an indirect but essential target group, as improved classification ultimately contributes to the development of more tailored and patient-centered technologies.

### Design and Development

#### Study Design

The first iteration of the taxonomy development involved a scoping review to identify and synthesize existing taxonomies and empirical studies focusing on AI-enabled epilepsy monitoring according to vom Brocke et al [30]. This scoping review was conducted in accordance with the Joanna Briggs Institute guidelines [31] and the Preferred Reporting Items for Systematic reviews and Meta-Analyses extension for Scoping Reviews (PRISMA-ScR) [32]. Search reporting follows PRISMA-S [33]. The completed PRISMA-S checklist is provided in Checklist 1, and full reproducible database-specific search strategies, including all update runs, are available in Multimedia Appendix 4.

#### Eligibility Criteria

We defined eligibility criteria following the Joanna Briggs Institute guidance for scoping reviews using the PCC (population, concept, and context) framework [31].

- Population: Studies involving human participants with epilepsy or epileptic seizures (or human seizure events captured on video) were included; animal studies were excluded.
- Concept: We included studies reporting AI-based or machine learning–based approaches for seizure monitoring, detection, classification, or prediction that used visual or video data (eg, computer vision–based monitoring). Studies focusing solely on EEG or wearable sensing without any visual or video component were excluded. We included only studies reporting the development and/or evaluation of a vision-based AI-ML system or algorithm for epilepsy monitoring. Studies describing rule-based approaches without an AI-ML component were excluded.
- Context: We considered any care context, including clinical environments (eg, epilepsy monitoring units and video-EEG settings), laboratory or sleep settings, and home or ambulatory monitoring. No restrictions were applied by country or geographic region.

Original empirical studies were included as primary evidence, whereas review articles were excluded. Review articles were excluded from the scoping review because the objective was to synthesize and map primary empirical evidence directly, thereby avoiding duplication of data and potential bias introduced by secondary interpretations. We included peer-reviewed, full-text research articles as eligible source types and excluded protocols, editorials, letters, commentaries, and abstract-only records. We restricted inclusion to English-language publications and to studies published from 2013 onward. This restriction aligns with the emergence of modern DL in computer vision and medical imaging. The breakthrough of deep CNNs in the 2012 ImageNet competition fundamentally changed image analysis and triggered the rapid expansion of DL approaches across domains, including medical imaging [34]. Given that our taxonomy aims to characterize current AI- and DL-based video monitoring approaches, restricting inclusion to studies published from 2013 onward is methodologically consistent with the technological evolution of the field.

#### Information Sources and Search Strategy

To identify relevant studies on AI-based monitoring systems for patients with epilepsy and visual data, the following search string was used to search in the title, abstract, and keywords: (“epilep*” OR “seizure*” OR “epileptic seizure*” OR “seizure disorder*” OR “tonic-clonic” OR “tonic clonic” OR “ictal” OR “convulsions” OR “generalized tonic-clonic” OR “grand mal”) AND (“AI” OR “artificial intelligence” OR “machine learning” OR “deep learning” OR “deep neural network*” OR “convolutional neural network*” OR “CNN”) AND (“predict*” OR “monitor*” OR “forecast*” OR “observ*” OR “recognit*” OR “alert*” OR “track”) AND (“video*” OR “camera*” OR “depth sensor*” OR “3D camera*” OR “vision-based” OR “vision based” OR “visual*” OR “motion*” OR “computer vision” OR “pose estimation”) AND PUBYEAR>2012. Search strategies were developed de novo and were not adapted from prior literature reviews. The information sources included Scopus (Elsevier), Web of Science Core Collection (Clarivate), and PubMed (National Library of Medicine). In PubMed, the search strategy was additionally complemented with relevant MeSH (Medical Subject Headings) terms, which are provided in Multimedia Appendix 4 together with the full database-specific search strategies. Each database was searched separately on its native platform (Scopus, Web of Science Core Collection, and PubMed); we did not conduct simultaneous multidatabase searching on a single platform. We did not search study registries. The searches were initially run on January 6, 2025. During revision, we updated the search by rerunning all database searches with a refined strategy on October 1 and on November 17, 2025. The final search was conducted on January 16, 2026, for all databases, covering publications from January 2013 onward. Each update reran the full search in all databases. No automated alerts were used. Newly retrieved records were deduplicated against the existing library prior to screening. No published search filters were used; we applied database limits for document type (article) and language (English) as reported in the database-specific strategies (Multimedia Appendix 4). The scoping review focused on peer-reviewed literature indexed in the selected databases; additional sources such as trial registries, preprint servers, or gray literature were not included. We did not hand-search websites, journal tables of contents, or conference proceedings. No protocol was registered. No additional data or studies were sought by contacting authors, experts, manufacturers, or others.

#### Study Selection and Data Charting

The search strategy was peer-reviewed by another information technology expert using the Peer Review of Electronic Search Strategies (PRESS) checklist [35] and revised accordingly. All records were exported to Microsoft Excel. Duplicates were removed in EndNote 2025 (Clarivate) using the multistep deduplication method described by Bramer et al [36] prior to screening. After deduplication, titles and abstracts were screened independently by 3 reviewers against the eligibility criteria. Potentially relevant records underwent full-text assessment. Discrepancies were resolved through discussion until consensus was reached. Comprehensive data extraction tables covering taxonomy dimensions are provided in Multimedia Appendix 5, while study-level characteristics are presented separately in Multimedia Appendix 6. Study-level characteristics were systematically charted using a structured Excel-based coding template (Multimedia Appendix 7).

#### Synthesis and Taxonomy Development Process

Before synthesis, extracted data were harmonized by standardizing terminology (eg, model types, evaluation metrics, and clinical settings). Consistent with scoping review guidance, critical appraisal of the individual sources of evidence was not performed. Given the heterogeneity of study designs and outcomes, no statistical pooling or meta-analysis was conducted; instead, findings were synthesized narratively and tabulated to highlight recurring dimensions, methodological trends, and research gaps.

The included studies were analyzed in the first iteration, following a C2E approach. Information from the original studies was used to derive meta-characteristics and foundational dimensions that shaped the conceptual basis of the taxonomy by incorporating insights from proof-of-concept and market-ready solutions. Three reviewers extracted data using the same structured Excel-based coding template (Multimedia Appendix 7), and discrepancies were resolved through discussion. The included studies were heterogeneous and frequently lacked components (eg, control groups and patient-level outcomes) necessary for structured bias assessment tools. Instead, we documented methodological limitations narratively and qualitatively considered transparency indicators to support credibility.

To integrate the perspective of potential end users, a *second iteration* was conducted using an E2C approach. The use of a Delphi study, as outlined by Gallego and Bueno [37], was particularly well suited to this work, enabling efficient consensus-building among a specialized group of domain experts within a limited time frame. This approach facilitated iterative refinement of the taxonomy while maintaining methodological rigor, ensuring that the resulting framework was both practically relevant and theoretically sound. By incorporating structured rounds of feedback, the process enhanced the validity of the taxonomy and aligned it with established quality criteria for taxonomy development. This Delphi study [37] was initiated with domain experts, including clinicians, engineers, and AI developers (Multimedia Appendix 8). Specifically, the panel consisted of AI and clinical experts from practice (P1, P2, and P3) and researchers in AI, eHealth, data security, and taxonomy development (R1, R2, R3, R4, R5, and R6). Their qualitative feedback led to the refinement of the taxonomy and the addition of 4 new dimensions. As a result, the ending conditions for taxonomy development were not yet met, and the process continued.

The *third iteration* returned to an inductive E2C approach. We accumulated the dimensions and characteristics for the subsequent iterations by searching for market-ready solutions of AI technologies for vision-based epilepsy monitoring to align with practice. A market analysis was conducted using the Crunchbase database to examine existing commercial systems. Crunchbase constitutes a leading internet database provider for business information, such as products, venture capital, and industries [38]. The search terms “epilepsy” and “video monitoring” identified only one company, “Neuro Event,” which developed the monitoring system “NELLI” [39]. In addition, the video-EEG monitoring system “SEER” was included in the analysis [24]. This step ensured that the taxonomy not only reflected academic research but also captured characteristics of real-world applications and emerging industry trends.

### Demonstration and Evaluation

Combining iterations 4 and 5 within this section is warranted by their shared method (E2C, Delphi study), common participants, and uniform data collection instruments. Both iterations operationalize the application of the taxonomy and its *ex-post* assessment. The focus shifts from validation of objective to subjective ending conditions. An integrated report better represents the iterative logic of the taxonomy development, ensures comparability across rounds, and provides a coherent audit trail for how the ending conditions were examined.

In the *fourth iteration*, a second round of the Delphi study [37] was conducted with the same group of experts. As part of this process, the experts received the iteratively developed taxonomy via email, including detailed descriptions of each dimension and characteristic as derived through the previous iterations. They were explicitly instructed to evaluate whether the identified dimensions and characteristics were both necessary and sufficient for classifying AI technologies in vision-based epilepsy monitoring and to determine if any essential aspects were missing, ambiguous, or misclassified. Furthermore, the experts were asked to systematically assess the taxonomy against the taxonomy design principles [26] to examine whether the objective ending conditions were fulfilled.

The *fifth iteration* involved conducting a third round of the Delphi study [37] with the same group of experts. They reviewed the updated taxonomy and provided structured feedback, particularly with regard to the subjective ending conditions [27]. These conditions include *conciseness*, *comprehensiveness*, *robustness*, *explainability*, and *extensibility*. To facilitate structured feedback, experts were provided with a standardized evaluation template that contained explicit questions for each subjective ending condition to elicit qualitative comments and suggestions related to each of these conditions. For *conciseness*, experts judged whether any dimension or characteristic was redundant or could be merged without loss of meaning. The c*omprehensiveness* of the taxonomy was investigated through its mapping to typical and rare use cases from vision-based epilepsy monitoring to identify potential gaps. *Robustness* was examined using deliberately atypical vignettes to evaluate whether classification guidance remained reliable under nonstandard conditions. *Explainability* was evaluated by having experts restate the taxonomy’s distinctions and indicate whether the aspects, dimensions, characteristics, and naming conventions supported coherent interpretation across stakeholders. *Extensibility* was assessed by requesting proposals for plausible future additions and checking whether these could be accommodated without reworking existing dimensions. Experts then issued an overall judgment on whether the subjective ending conditions were met, with a short rationale. Aligned with the *evaluation goal* of establishing the taxonomy’s *usefulness* for health care professionals, researchers, and technology developers, we implemented a summative, ex-post usefulness check within the Delphi study. The same group of experts mapped the taxonomy to typical and edge-case scenarios drawn from vision-based epilepsy monitoring practice and reflected on its applicability for conducting analyses, enabling systematic comparisons, structuring communication across roles, and informing design decisions. Responses were captured within a structured template that elicited brief rationales and judgments of sufficiency [27].

In summary, experts provided a brief overall statement on whether the objective and subjective ending conditions were met, alongside a justification of the usefulness of the taxonomy. The outcomes of these judgments are reported in the *Results* section.

### Communication

Reporting the taxonomy through a frequency analysis shows which fields are well served and where the gaps are located. To communicate the state of the field relative to the taxonomy, we conducted a descriptive frequency analysis consistent with step 6 *Report Taxonomy* of our methodological approach [27]. The unit of analysis was the set of studies included in the scoping review and the results of the market analysis for AI technologies for vision-based epilepsy monitoring solutions. A standardized coding template derived from the final taxonomy guided the extraction of aspect, dimension, and characteristic labels. Using a predefined matrix, 3 researchers independently annotated each study for the presence of characteristics within every characteristic, allowing multilabel coding where the taxonomy permits (eg, multiple data acquisition sources) and otherwise enforcing exactly one characteristic per dimension. Ambiguities and missing information were marked as not reported following explicit decision rules, with disagreements resolved by consensus after calculating interrater agreement. We then aggregated binary indicators to produce per-characteristic frequencies at the level of studies and, where available, at the level of distinct systems. The resulting counts were summarized in tabular form and visualized to communicate coverage across dimensions and characteristics.

### Ethical Considerations

This work did not involve medical products or medicinal trials. It combines a Delphi study and a scoping review. According to the guidelines of the research committee of the University of Osnabrück (Germany), scoping reviews do not require ethical approval. Furthermore, the Research Ethics Committee of the University of Osnabrück (Germany) determined that the Delphi study does not constitute human subjects research (decision/date: NHSR/19.09.2025). This Delphi study focused only on professional opinions from adult participants and involved anonymous expert elicitation and no collection of identifiable private information. Therefore, formal ethics approval was not required. Nevertheless, all panelists received written information about the study purpose (including aims, procedures, risks, and data handling) and provided written informed agreement to participate. Participation was voluntary and could be discontinued at any time without penalty. No vulnerable populations were included. All Delphi panelists were adults (aged ≥18 y) and participated voluntarily after receiving information about the study’s aims, procedures, risks, and data handling. No clinical information or identifiable patient data were collected. Expert feedback was documented using coded identifiers and analyzed in aggregate. No direct personal identifiers are reported. All files were kept on a secure institutional platform of the University Osnabrück with access restricted to the study team with password-protected login and handled in compliance with the General Data Protection Regulation. Participants received no financial or material compensation for taking part in this work.

## Results

### Study Characteristics

#### Selection of Sources of Evidence

**Figure 2.**
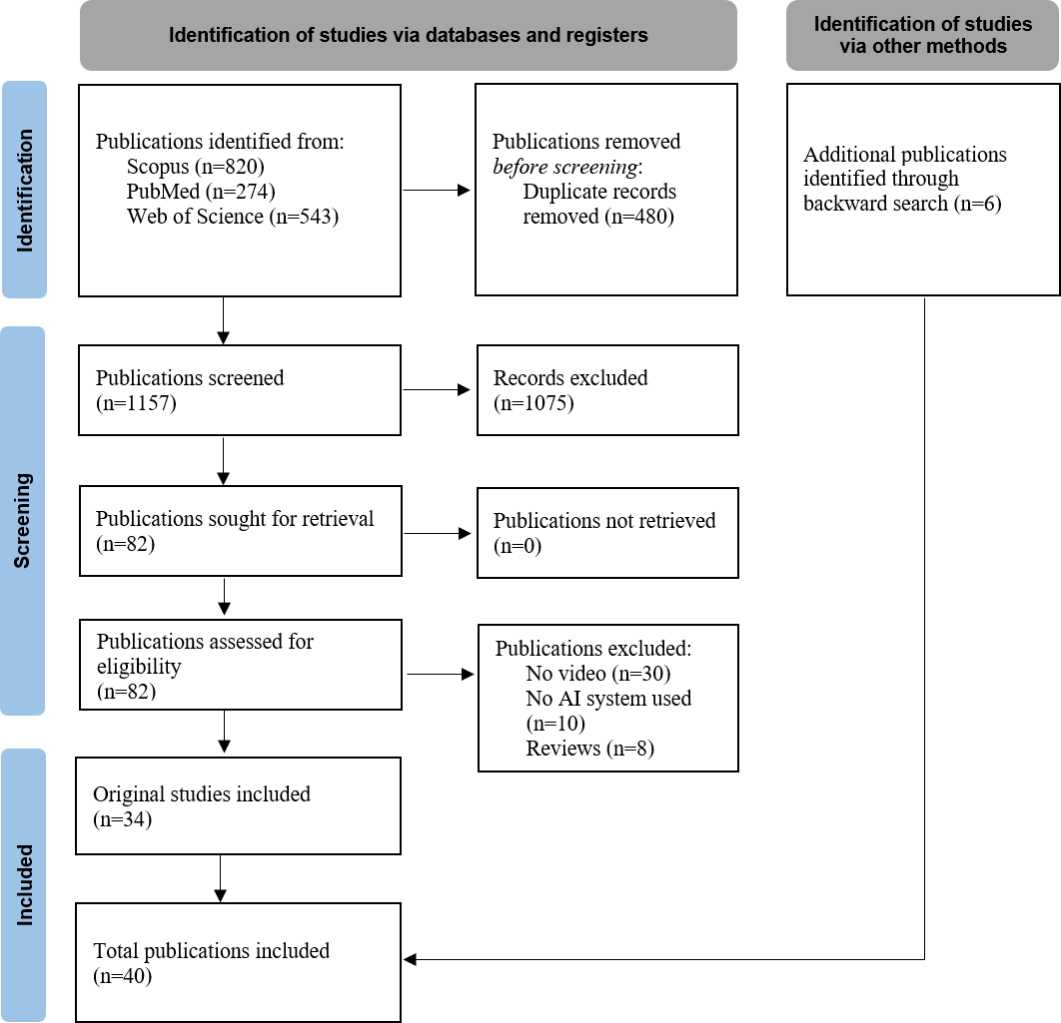
PRISMA (Preferred Reporting Items for Systematic Reviews and Meta-Analyses) flowchart of the study selection process. AI: artificial intelligence.

After the removal of duplicates, this yielded a total of 1157 hits in Scopus, PubMed, and Web of Science databases. Three authors screened all titles and abstracts independently for eligibility, followed by full-text assessment of eligible records. Discrepancies were resolved through discussion. No automation tools were used during screening or data charting. Following a comprehensive scoping review, 30 publications were excluded due to the absence of video data, and 10 papers were excluded because they did not involve any AI technologies. In addition, 8 review articles (secondary research) were excluded. After screening titles, abstracts, and reading full texts, 40 original studies remained for dimension and characteristic analysis (Figure 2). Three authors collected data from the reports and assessed each study independently. No automation tool was used. On the basis of the scoping review, we initially identified the first categories of the taxonomy analysis: tracking target, period of epilepsy, type of classifier, evaluation metrics, scope, and image processing. The selection process is summarized in the PRISMA flow diagram (Figure 2).

#### Characteristics of Sources of Evidence

Key study characteristics are transparently charted across the included evidence, enabling comparison of setting, study design, evaluation maturity, data source, sample type, age group, and sample size (Table 1). All extraction study details are provided in Multimedia Appendix 9. Across the original studies (N=40), the evidence base is predominantly situated in hospital wards (n=30, 75%), follows method development designs (n=28, 70%), and reports pilot evaluations in real-world settings (n=32, 80%). Retrospective data use (n=23, 57,5%) is more common than prospective designs, and participant samples are generally small, with most studies including 50 or fewer individuals. Overall, the evidence landscape reflects a field centered on technical feasibility and early-stage validation within controlled clinical environments. These structural patterns informed the subsequent taxonomy development by highlighting recurring dimensions (eg, setting, evaluation maturity, and data source type) and revealing systematic gaps that required explicit conceptual representation within the classification framework.

**Table 1.** Study characteristics.

Dimension and characteristic	Sources, n (%)
Setting	
Hospital ward	30 (75)
Home	6 (15)
Simulated	2 (5)
Residential care	2 (5)
Study design	
Method development	28 (70)
Feasibility	6 (15)
Observational study	4 (10)
Dataset development	2 (5)
Evaluation maturity	
Pilot in real setting	32 (80)
Laboratory prototype	6 (15)
Deployed	2 (5)
Data source type	
Retrospective	23 (57.5)
Prospective	11 (27.5)
Staged	3 (7.5)
Mixed	2 (5)
Public dataset	1 (2.5)
Sample type	
Patients	38 (95)
Healthy volunteers	2 (5)
Age group	
Adults	32 (80)
Pediatric	7 (17.5)
Mixed	1 (2.5)
Participant sample size (total N)	
≤10 (very small)	12 (30)
11‐50 (small)	19 (47.5)
51‐200 (medium)	4 (10)
>200 (large)	4 (10)
Not reported	1 (2.5)

#### Synthesis of Results

**Figure 3.**
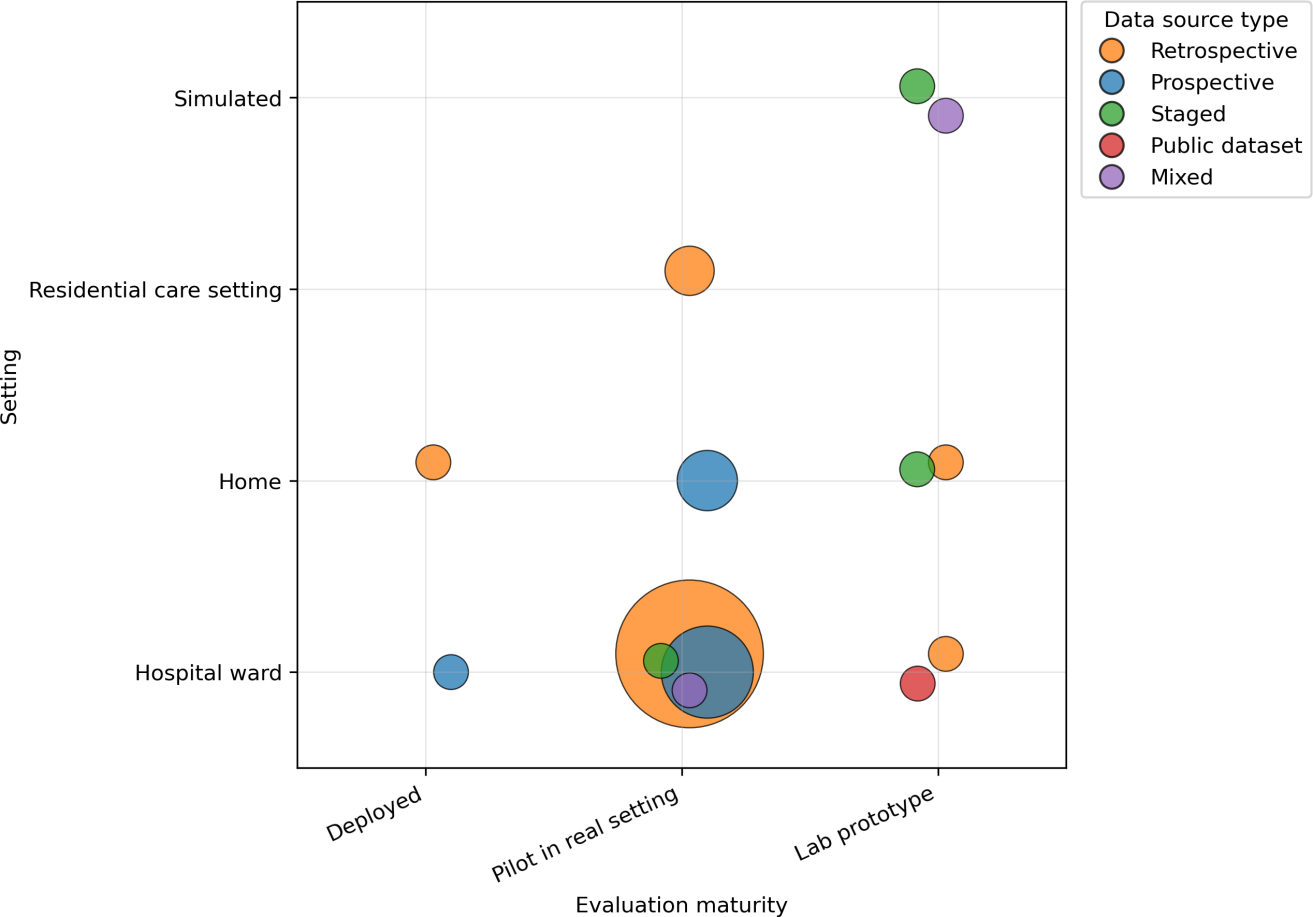
Evidence map of the study results by setting and evaluation maturity.

To support a structured overview of the evidence base and make gaps visible at a glance, we created an evidence map to visualize the distribution of evidence across setting and evaluation maturity (bubble plot; Multimedia Appendix 10). The map highlights 3 recurring gaps: first, prospective real-world evaluations in home settings remain scarce (Figure 3); second, the literature focuses predominantly on detection and classification, while prediction is comparatively underrepresented (Multimedia Appendix 10); and third, heterogeneous and incomplete reporting—particularly regarding classifier type and the use of standardized performance metrics—limits cross-study comparability and makes it difficult to judge methodological maturity and practical readiness (Multimedia Appendix 10).

Taken together, these gaps indicate that the field is not only uneven in where and how systems are evaluated but also in what is targeted and how results are reported. To move beyond gap identification and provide a structured synthesis that can guide future development, evaluation, and reporting, the next section derives a taxonomy from the included sources. This taxonomy consolidates the observed design and evaluation characteristics into a coherent classification scheme and explicitly incorporates the underrepresented areas identified in the evidence map.

### Overview of All Taxonomy Iterations

To ensure a transparent and comprehensible presentation of the taxonomy development process, this section provides an overview of the 5 iterative cycles undertaken to derive the final taxonomy. Each iteration contributed incrementally by identifying, refining, or validating dimensions and characteristics, thereby systematically constructing a robust framework for classifying AI technologies in vision-based epilepsy monitoring.

Developed through an iterative methodology, the final taxonomy consists of 23 dimensions and 102 characteristics, capturing the complexity and heterogeneity of current and emerging solutions in this field. In Multimedia Appendix 3, we provide a schematic overview of the iterations and the dimensions identified at each stage, illustrating the evolution of the taxonomy and the structured expansion of its scope.

During the first iteration, we initially identified 16 dimensions: scope [40], target group [22], period of epilepsy [41,42], data acquisition source [43,44], tracking target [45], video tracking [46,47], image processing [14], type of classifier [42,48], performance metrics [41,49], environment [50], seizure classification [51], medical device [17], salient attributes [5], data privacy [18], user interface [45], and user interaction [3]. These newly uncovered dimensions broadened the taxonomy to better reflect the diverse technical and contextual facets of AI-enabled visual monitoring solutions. As the predefined ending conditions [26] had not yet been fulfilled, iteration 2 used an E2C approach, leveraging a Delphi study to integrate expertise from researchers and practitioners. This strategy led to the addition of 4 further dimensions: cryptographic measure, communication mode, response type, and information purpose. The structured expert feedback not only ensured practical relevance but also helped maintain methodological rigor aligned with established quality criteria for taxonomy development. Despite these advancements, the ending conditions remained unmet, leading to iteration 3. In this stage, practical alignment was prioritized by conducting a market analysis of commercial solutions using Crunchbase and targeted internet research. As a result, 3 new dimensions were identified: computing paradigm, connection type, and support [24,39]. This practical focus ensured that the taxonomy adequately captured real-world implementation aspects and industry trends. The ending conditions were not yet met; another iteration followed. Finally, iterations 4 and 5 consisted of a second round of the Delphi study with the same panel of domain experts to rigorously evaluate the taxonomy against both the objective and subjective ending conditions in accordance with Nickerson et al [26]. The experts confirmed the taxonomy’s conciseness, robustness, explainability, comprehensiveness, and extensibility, with no additional dimensions proposed.

### Final Taxonomy

#### Overview

The defined taxonomy met all predefined ending conditions after 5 iterations of developing the aspects, dimensions, and characteristics. Finally, the subjective ending conditions, including conciseness, robustness, explainability, comprehensiveness, and extensibility, were fulfilled. It is imperative to note that the completion of 5 subjective ending conditions indicates a high sufficiency of the taxonomy. However, we present the final outcome of our research: Established through a rigorous iterative methodology, the taxonomy comprises 6 overarching aspects, encompassing 23 dimensions and a total of 102 characteristics. Each aspect integrates specific dimensions and associated characteristics, offering a rigorous foundation for evaluating the functionality and applicability of these systems (Table 2). The target audience of this taxonomy includes clinical researchers, neurologists, health care professionals, decision-makers, and developers who aim to evaluate, design, or implement AI technologies in vision-based epilepsy monitoring.

**Table 2.** Final taxonomy of emerging artificial intelligence (AI) technologies in vision-based epilepsy monitoring.

Aspect and dimension	Characteristics
Application and context	
Scope	Detection Classification Prediction
Target group	Patients with epilepsy Caregivers Medical professionals Data scientists
Environment	Stationary Mobile
Seizure classification	Nonmotor symptoms Motor symptoms
Period of epilepsy	Interictal Preictal Ictal Postictal
Data acquisition source	Depth sensors Infrared 2D camera 3D camera Video-EEG^a^ ECG^b^ Audio
Visual analysis	
Tracking target	Body Face Sleeping area Room overview
Video tracking	MD^c^ BC^d^ MOI^e^ ROI^f^ HMD^g^ SKS^h^ Appearance- and feature-based methods
Image processing	Optical flow Frame differencing Spatiotemporal interest points Contrast-based analysis
AI model	
Type of classifier	SVM^i^ RF^j^ MP^k^ CNN^l^ GMM^m^ LSTM^n^ I3D^o^ Other types of classifier
Performance metrics	Accuracy Sensitivity Specificity *F*_1_-score Precision Recall False-positive rate Area under the curve
Market identity	
Medical device	Certified Not certified Proof of concept
Salient attribute	Environmental robustness Cost-efficiency Real-time analysis Ease of use High system performance
Data privacy	Anonymization Pseudonymization No privacy preserving measures Synthetic data
Cryptographic measure	Encryption in transit Encryption at rest No encryption
System architecture	
User interface	Web platform Voice assistant Mobile app Desktop app Wearable device
User interaction	Reporting Interactive Adaptive No interaction
Computing paradigm	Cloud-based platform Edge-based platform Local on device
Connection type	Wi-Fi Built-in-modem Ethernet Bluetooth
Support	On call System setup Help center Daily technical checks Expert data review Chat
Feedback system	
Communication mode	Real time Periodic Event based On demand
Response type	Visual Auditory Haptic Text based
Information purpose	Alerting or warning Performance evaluation Recommendation User learning

a EEG: electroencephalogram.

b ECG: electrocardiogram.

c MD: movement dynamics.

d BC: biomechanical characteristics.

e MOI: movement of interest.

f ROI: region of interest.

g HMD: head movement detection.

h SKS: simple keypoint system.

i SVM: support vector machine.

j RF: random forest.

k MP: multilayer perceptron.

l CNN: convolutional neural network.

m GMM: Gaussian mixture model.

n LSTM: long short-term memory.

o I3D: inflated 3D.

#### Application and Context

The first aspect delineates the operational domain and intended purpose of AI technologies in vision-based epilepsy monitoring. It considers their primary scope ranging from seizure detection, classification, and prediction [8,13] to the specific target group they serve, including patients with epilepsy, caregivers, medical professionals, and data scientists [3,45]. This aspect further differentiates systems based on their environment, distinguishing between stationary implementations, such as hospital- and home-based systems with mobile configurations that allow for monitoring in diverse environments [3,45]. Moreover, it incorporates distinctions in seizure classification, differentiating between motor and nonmotor symptomatology [23]. The temporal dimension of epilepsy monitoring is also captured, classifying the period of epilepsy as interictal, preictal, ictal, or postictal [8]. Additionally, it accounts for various data acquisition modalities, including depth sensors, infrared imaging, 2D and 3D cameras, video-EEG, electrocardiography, and audio [23,52].

#### Visual Analysis

The second aspect examines the computational techniques used to track and interpret seizure-related phenomena. This aspect defines key tracking targets, encompassing body, face, sleeping area, and room overview [3,47,52,53]. It further classifies video tracking methodologies, including movement dynamics [17,50,51,54], biomechanical characteristics [54,55], movement of interest [5,45,56], region of interest [16,56-58], head movement detection (HMD) [55,59], simple keypoint system [47,59], and appearance- and feature-based methods leveraging visual attributes [14,54]. Furthermore, it includes image processing techniques such as optical flow [6,16,60-62], frame differencing [63], spatiotemporal interest points [57,62], and contrast-based analysis [6,57,60], all of which contribute to enhancing the accuracy and robustness of seizure identification [6,23].

#### AI Model

The third aspect addresses the algorithmic paradigms underpinning epilepsy monitoring systems. This aspect details the types of classifier, including support vector machine [48,64], random forest [48], multilayer perceptron [48,59], CNN [6,50,54,57,61,65], Gaussian mixture model [63], long short-term memory (LSTM) [50,53,57,61,65], inflated 3D [18,54,66], and other types of classifiers, for example, automated seizure and spike detection [10]. An essential topic of this aspect is the assessment of system efficacy using well-established performance metrics, including accuracy, sensitivity, specificity, *F*_1_-score, precision, recall, false-positive rate, and area under the curve [8,12,23,67,68].

#### Market Identity

The fourth aspect captures regulatory, commercial, and privacy-related considerations. It classifies systems based on their certification status, differentiating between certified [45] and not certified systems and proof-of-concept systems [48,60,61,65]. It also identifies salient attributes, such as environmental robustness, cost-efficiency, real-time analysis [55,62], ease of use [16], and high system performance [62]. Additionally, it examines data privacy protocols, distinguishing among anonymization, pseudonymization, no privacy preserving measures (eg, no anonymization or pseudonymization) [14,22,68], and synthetic data (R1 and R4) while also addressing cryptographic security measures, including encryption in transit, encryption at rest (R3), and no encryption [18,46]. Privacy-preserved detection and new frameworks are on the rise [69].

#### System Architecture

The fifth aspect encompasses the technical structure and interaction modalities of AI technologies in vision-based epilepsy monitoring. This aspect categorizes user interfaces ranging from web platforms [3,45], voice assistants (P1 and R2), mobile and desktop apps, and wearable devices [3,10,16]. It also distinguishes user interaction paradigms, including reporting, interactive [3,10], adaptive (R4 and R5), and no interaction because systems only track patients without involving an interaction opportunity directly for this target group, for example, the system extracts interesting video sequences and saves and uploads these for the medical supervisor [17]. Computing paradigm is classified as cloud-based platform, edge-based platform, and local on device [22,46,67,68], while connection types are categorized into Wi-Fi, built-in modem, Ethernet, and Bluetooth [24,39]. Furthermore, this aspect considers system support, such as on call, system setup, help center, daily technical checks, expert data review, and chat [24,39].

#### Feedback System

The sixth and final aspect pertains to mechanisms for delivering feedback to users. These dimensions represent topics that are more forward-looking and have not yet been fully established in research or practice (Table 3). This aspect categorizes communication mode based on real-time, periodic, event-based, and on-demand feedback (R1, R2, and R4). It also classifies response type as visual, auditory, haptic, or text-based (R2 and R4). The information purpose marks the last characteristic and describes diverse functional objectives, including alerting and warning users, performance evaluation, recommendations, and user learning to enhance self-management (R2). On the basis of feedback, these elements were included to reflect potential future developments and ensure that the taxonomy remains relevant over time. By incorporating this forward-looking perspective, the taxonomy can serve as a foundation for both current classification and future advancements of AI technologies in vision-based epilepsy monitoring.

**Table 3.** Frequency analysis based on the scoping review (n=0-40).

Aspect and dimension	Characteristics
Application and context	
Scope	Detection (n=34, 85%) Classification (n=24, 60%) Prediction (n=2, 5%)
Target group	Patients with epilepsy (n=8, 20%) Caregivers (n=2, 5%) Medical professionals (n=33, 82.5%) Data scientists (n=17, 42.5%)
Environment	Stationary (n=35, 87.5%) Mobile (n=7, 17.5%)
Seizure classification	Nonmotor symptoms (n=8, 20%) Motor symptoms (n=37, 92.5%)
Period of epilepsy	Interictal (n=9, 22.5%) Preictal (n=3, 7.5%) Ictal (n=37, 92.5%) Postictal (n=3, 7.5%)
Data acquisition source	Depth sensors (n=9, 22.5%) Infrared (n=20, 50%) 2D camera (n=36, 90%) 3D camera (n=5, 12.5%) Video-EEG^a^ (n=21, 52.5%) ECG^b^ (n=3, 7.5%) Audio (n=6, 15%)
Visual analysis	
Tracking target	Body (n=30, 75%) Face (n=13, 32.5%) Sleeping area (n=23, 57.5%) Room overview (n=2, 5%)
Video tracking	MD^c^ (n=30, 75%) BC^d^ (n=1, 2.5%) MOI^e^ (n=16, 40%) ROI^f^ (n=17, 42.5%) HMD^g^ (n=3, 7.5%) SKS^h^ (n=8, 20%) Appearance and feature-based methods (n=31, 77.5%)
Image processing	Optical flow (n=13, 32.5%) Frame differencing (n=3, 7.5%) Spatiotemporal interest points (n=6, 15%) Contrast-based analysis (n=11, 27.5%)
AI^i^ model	
Type of classifier	SVM^j^ (n=8, 20%) RF^k^ (n=2, 5%) MP^l^ (n=4, 10%) CNN^m^ (n=22, 55%) GMM^n^ (n=3, 7.5%) LSTM^o^ (n=11, 27.5%) I3D^p^ (n=4, 10%) Other types of classifier (n=13, 32.5%)
Performance metrics	Accuracy (n=20, 50%) Sensitivity (n=25, 62.5%) Specificity (n=16, 40%) *F*_1_-score (n=14, 35%) Precision (n=15, 37.5%) Recall (n=8, 20%) False-positive rate (n=12, 30%) Area under the curve (n=13, 32.5%)
Market identity	
Medical device	Certified (n=6, 15%) Not certified (n=0, 0%) Proof of concept (n=34, 85%)
Salient attribute	Environmental robustness (n=4, 10%) Cost-efficiency (n=4, 10%) Real-time analysis (n=3, 7.5%) Ease of use (n=4, 10%) High system performance (n=3, 7.5%)
Data privacy	Anonymization (n=7, 17.5%) Pseudonymization (n=2, 5%) No privacy-preserving measures (n=0, 0%) Synthetic data (n=0, 0%)
Cryptographic measure	Encryption in transit (n=3, 7.5%) Encryption at rest (n=0, 0%) No encryption (n=2, 5%)
System architecture	
User interface	Web platform (n=6, 15%) Voice assistant (n=0, 0%) Mobile application (n=4, 10%) Desktop application (n=3, 7.5%) Wearable device (n=1, 2.5%)
User interaction	Reporting (n=6, 15%) Interactive (n=10, 25%) Adaptive (n=0, 0%) No interaction (n=1, 2.5%)
Computing paradigm	Cloud-based platform (n=7, 17.5%) Edge-based platform (n=2, 5%) Local on device (n=14, 35%)
Connection type	Wi-Fi (n=2, 5%) Built-in-modem (n=1, 2.5%) Ethernet (n=3, 7.5%) Bluetooth (n=1, 2.5%)
Support	On call (n=1, 2.5%) System setup (n=3, 7.5%) Help center (n=0, 0%) Daily technical checks (n=3, 7.5%) Expert data review (n=3, 7.5%) Chat (n=0, 0%)
Feedback system	
Communication mode	Real time (n=2, 5%) Periodic (n=2, 5%) Event-based (n=3, 7.5%) On demand (n=5, 12.5%)
Response type	Visual (n=4, 10%) Auditory (n=1, 2.5%) Haptic (n=0, 0%) Text-based (n=4, 10%)
Information purpose	Alerting and warning (n=2, 5%) Performance evaluation (n=4, 10%) Recommendation (n=0, 0%) User learning (n=0, 0%)

a EEG: electroencephalogram.

b ECG: electrocardiogram.

c MD: movement dynamics.

d BC: biomechanical characteristics.

e MOI: movement of interest.

f ROI: region of interest.

g HMD: head movement detection.

h SKS: simple keypoint system.

i AI: artificial intelligence.

j SVM: support vector machine.

k RF: random forest.

l MP: multilayer perceptron.

m CNN: convolutional neural network.

n GMM: Gaussian mixture model.

o LSTM: long short-term memory.

p I3D: inflated 3D.

### Demonstration and Evaluation

All 9 experts expressed explicit satisfaction with the taxonomy’s scope and structure, confirming that it was well aligned with both theoretical rigor and practical relevance. Every identified object was examined, each characteristic classified at least one object, no further dimensions or characteristics were introduced, and cell combinations were unique and nonredundant, thereby fulfilling the objective ending conditions in iteration 4 and concluding the taxonomy development process. In line with the *Introduction*’s stated purpose (why, how, and what), these results complete the formative, *ex-ante* stage of the *ETDP* and motivate a summative check of utility.

Building on this confirmation, the same expert group proceeded to appraise whether the taxonomy is concise, comprehensive, robust, explainable, and extensible for its intended use in iteration 5 to evaluate the subjective ending conditions. Panelists reported that the taxonomy is *concise*, noting an absence of redundant elements while allowing for focused extensions was helpful. Experts judged the taxonomy as *comprehensive* and the coverage to be largely sufficient for the domain (P1-P3 and R1-R6). One expert described the final taxonomy as “very comprehensive and complete in the categories considered” (R1). Furthermore, experts indicated that guidance remains reliable for nonstandard or edge scenarios, characterizing *robustness* as good and emphasizing maintained flexibility and precision (P1-P3 and R1-R6). The experts reported that the taxonomy’s structure supports understanding; distinctions were seen as transparent and traceable, and the systematic differentiation was said to improve *explainability* and provide a comprehensive overview (P1-P3 and R1-R6). It is acknowledged that certain terminology may not be comprehensible to specialists. For instance, medical terminology may not be readily accessible to IT professionals, and conversely, IT terminology may not be readily comprehensible to medical staff. The incorporation of a detailed description or an extended legend accompanied by comprehensive explanations could prove beneficial in this context (R1 and R6). Facing the condition of *extensibility*, the forward compatibility was consistently affirmed. Experts noted that, due to its modular structure, the taxonomy can be extended by adding elements without rewriting existing ones (P1-P3 and R1-R6). In aggregate, experts assessed the subjective ending conditions as fulfilled, indicating that the taxonomy is both theoretically coherent and practically usable for its intended purposes.

Experts judged the taxonomy to be *useful* for its intended target group and purpose (P1-P3 and R1-R6). Panelists provided convergent evidence that the taxonomy is practically useful for analysis, comparison, communication, and decisions. They noted that, in clinical settings, the taxonomy functions as a common language for appraising systems and coordinating with nonclinical stakeholders, ultimately aiding selection and configuration. Clinicians reportedly view it as a shared frame that supports systematic comparison of alternatives and clearer stakeholder communication, thereby informing practical choices in deployment and workflow integration. “This taxonomy could facilitate a systematic analysis of the landscape of AI-based epilepsy monitoring and drive targeted research and development in areas where it is most required,” declares one expert (R5). Researchers indicated that the taxonomy enables structured synthesis and comparability of studies, facilitates the identification of gaps, and provides a stable basis for cumulative evidence building. From a clinical standpoint, experts reported that the taxonomy offers a common structure for evaluating candidate systems and coordinating expectations among care teams and technology partners, which, in turn, supports context-appropriate choices (R4). From a research perspective, it was described as enabling consistent coding, comparison, and aggregation of evidence, while surfacing neglected areas for future work (R2 and R4). In development contexts, experts indicated that it makes market and clinical requirements more transparent and helps align design trade-offs with medical preconditions (R4 and R6). From an industry perspective, experts indicated that the taxonomy clarifies domain constraints and medical prerequisites, translating needs into design requirements and product positioning (R4 and R6). One expert highlighted that the clear delineation of categories creates shared understanding and a robust foundation for decision-making (R1), whereas another underscored that such structure can advance work in areas with the greatest unmet need (R2). Overall, experts characterized usefulness as high and emphasized that the taxonomy’s clear demarcations promote shared understanding and provide a dependable decision base (P1-P3 and R1-R6).

Viewed in its entirety, the evaluation examined whether the taxonomy is sufficient, clear, applicable, and extensible for its intended use. Complementing the formative, ex-ante checks applied during construction (objective and subjective ending conditions across iterative C2E and E2C cycles), the Delphi study offered an ex-post assessment by domain experts. Their consensus confirmed that the taxonomy’s scope and structure are well aligned with the field and that no further dimensions were required (P1-P3 and R1-R6). This step closes the ETDP loop regarding the demonstration and evaluation phase. The developed taxonomy is theoretically coherent and practically usable for classifying AI technologies in vision-based epilepsy monitoring and for supporting consistent study design and reporting.

### Communication: Frequency Analysis

As demonstrated in iteration 3 of the practical assessment, the market analysis revealed that many systems have not yet reached market readiness. Given this constrained evidence base, we included one real-world application as an illustrative use case rather than as the basis for a comparative market analysis. Specifically, we provide a detailed classification example of the market-ready system NELLI (Multimedia Appendix 11) [39]. As the number of real-world applications was insufficient for robust market-level inference, the subsequent synthesis focused on the scoping review evidence. To synthesize insights and highlight gaps, we conducted a frequency analysis across the studies of the scoping review (Multimedia Appendix 7). The analysis included 40 studies with multiple coding permitted per dimension when single studies implemented more than one approach. Therefore, frequencies represent counts of appearances rather than mutually exclusive proportions. The corresponding cases are reported transparently below (Table 3).

The aspect of *application and context* for AI technologies in vision-based epilepsy monitoring reveals the predominant aims to be detection (n=34, 85%) and classification (n=24, 60%), while prediction is almost absent (n=2, 5%). This pattern underscores a focus on immediate recognition of ictal events consistent with clinical priorities, whereas predictive systems remain an open area, likely reflecting their higher methodological complexity. Multiple entries for detection and classification reflect multiscope designs within the same study [17,18,55,56,62]. Target group analysis shows that the overwhelming majority of technologies are tailored to medical professionals (n=33, 82.5%) and secondly to data scientists (n=17, 42.5%), with only marginal attention given to patients with epilepsy themselves (n=8, 20%) or caregivers (n=2, 5%). This suggests a prevailing research trend toward supporting diagnostic and treatment processes within clinical workflows while largely neglecting patient-centered or caregiver-focused designs. The environmental context predominantly involves stationary settings (n=35, 87.5%) compared to mobile scenarios (n=7, 17.5%), reflecting the common use of video-EEG laboratories and controlled observation environments. Similarly, seizure classification heavily favors motor symptoms (n=37, 92.5%) over nonmotor symptoms (n=8, 20%), likely because motor phenomena are more easily captured via visual data, whereas nonmotor events often require multimodal sensing or subjective reporting. Regarding the period of epilepsy, the ictal phase is the principal focus (n=37, 92.5%), followed by preictal (n=3, 7.5%) and postictal (n=3, 7.5%) phases, with limited attention to interictal periods (n=9). This underlines a current emphasis on real-time seizure monitoring over long-term interictal assessment. The data acquisition landscape shows a diverse application of technologies: depth sensors (n=9, 22.5%), infrared (n=20, 50%), 2D cameras (n=36, 90%), 3D cameras (n=5, 12.5%), and video-EEG (n=21, 52.5%) are all well represented. Multimodal systems based on electrocardiography (n=3, 7.5%) and audio (n=6, 15%) are far less frequent, indicating that more multimodal approaches are necessary. Multiple entries for data sources arise when studies use several modalities concurrently [18,42,56,66,70,71].

The *visual analysis* also highlights the clear dominance of body-focused tracking (n=30, 75%) and sleeping area targeting (n=23, 57.5%), which likely reflects the typical contexts of nocturnal seizure detection. Multiple entries occur where similar events span body and sleeping area [44,60,70]. Face tracking (n=13, 32.5%) appears as a secondary priority, while room-wide overviews (n=2, 5%) are notably rare. For video tracking operators, most studies use movement dynamics (n=30, 75%), and many adopt appearance or feature-based pipelines (n=31, 77.5%). Movement of interest is reported in 16 studies (n=16, 40%), region of interest is reported in 17 studies (n=17, 42.5%), head movement detection appears in 3 studies (n=3, 7.5%), simple keypoint system in 8 studies (n=8, 20%), and biomechanical characteristics in 1 study (n=1, 2.5%). Multiple entries were made where single studies combined several operators [40,47,54,55]. For image processing, optical flow is comparatively common (n=13, 32.5%), followed by contrast-based analysis (n=11, 27.5%), whereas frame differencing (n=3, 7.5%) and spatiotemporal interest points (n=6, 15%) are used infrequently.

Within the aspect of *AI model*, CNNs are the most used classifiers (n=22, 55%), closely followed by LSTMs (n=11, 27.5%), with other models such as support vector machine (n=8), inflated 3D (n=4, 10%), multilayer perceptron (n=4, 10%), Gaussian mixture model (n=3, 7.5%), and random forest (n=2, 5%) used considerably less. This trend underscores the preference for DL approaches suited for complex spatiotemporal patterns in video data. In this dimension, multiple entries were acceptable because of multiple approaches in studies within AI models based on, for example, LSTM and CNN [43,44,51,65]. Performance evaluation predominantly relies on sensitivity (n=25, 62.5%), accuracy (n=20, 50%), specificity (n=16, 40%), precision (n=15, 37.5%), area under the curve (n=13, 32.5%), and *F*_1_-score (n=14, 35%), while more nuanced metrics such as recall (n=8, 20%) and false-positive rates (n=12, 30%) are less consistently reported. This points to a need for more comprehensive benchmarking that captures the practical trade-offs between detection sensitivity and false alarm rates.

*Market identity* is dominated by proof-of-concept solutions (n=34, 85%), with relatively few certified medical devices (n=6, 15%). Reported salient attributes cluster around environmental robustness (n=4, 10%), cost-efficiency (n=4, 10%), real-time analysis (n=3, 7.5%), ease of use (n=4, 10%), and high system performance (n=3, 7.5%). Privacy and security remain underreported. Anonymization (n=7, 17.5%) and pseudonymization (n=2, 5%) are occasionally specified; synthetic data (n=0, 0%) and explicit no privacy preserving measure (n=0, 0%) do not appear. Encryption in transit is sometimes stated (n=3, 7.5%), whereas encryption at rest (n=0, 0%) is not, and no encryption (n=2, 5%) is rarely reported.

Reporting on *system architecture* is uneven. User interfaces include web platforms (n=6, 15%), mobile apps (n=4, 10%), desktop apps (n=3, 7.5%), and wearables (n=1, 2.5%), with voice assistants (n=0, 0%). User interaction is mainly interactive (n=10, 25%) and reporting (n=6, 15%), less with no interaction (n=1, 2.5%), and adaptive interactions are absent (n=0, 0%). Computing paradigms split between cloud-based (n=7, 17.5%) and local on device (n=14, 35%), with edge-based platform (n=2, 5%). The connection type is rarely specified with Wi-Fi (n=2, 5%), built-in modem (n=1, 2.5%), Ethernet (n=3, 7.5%), and Bluetooth (n=1, 2.5%). Support modalities show system setup (n=3), daily technical checks (n=3, 7.5%), expert data review (n=3, 7.5%), and on call (n=1, 2.5%), but help center and chat are both absent (n=0, 0%).

To complete the results, the *feedback system* indicates that most implementations are geared toward retrospective review rather than immediate intervention. Communication modes are led by on-demand use (n=5, 12.5%), with periodic and event-based configurations occurring less frequently (each n=2, 5%), and true real-time communication remaining uncommon (n=2, 5%). Response types are dominated by visual and text-based outputs (each n=4, 10%), while auditory responses are rarely reported (n=1, 2.5%) and haptic feedback is absent (n=0, 0%). In terms of purpose, the literature primarily documents performance evaluation (n=4, 10%), with comparatively few systems supporting alerting or warning (n=2, 5%) and none providing recommendation or user-learning functions (each n=0).

This frequency analysis provides a structured overview of the current landscape of vision-based AI technologies for epilepsy monitoring and highlights prevailing research emphases as well as underexplored areas. By quantifying recurring dimensions and characteristics, this analysis complements the taxonomy by offering an additional empirical perspective on where the field currently concentrates its efforts and where evidence remains sparse. Beyond summarizing patterns, the frequency analysis increases the practical utility of the taxonomy by enabling systematic comparison across studies and supporting identification of research gaps. In practical terms, the resulting overview can support multiple stakeholder groups: researchers may use it to classify and contrast approaches and to prioritize future investigations, clinicians and decision-makers may use it to appraise the maturity and fit of available technologies for specific care contexts, and developers or companies may use it to benchmark solution profiles and identify opportunities for product development.

## Discussion

### Principal Findings

In this scoping review of vision-based AI technologies for epilepsy monitoring, we synthesized the evidence into a comprehensive taxonomy structured around application and context, visual analysis, AI model, system architecture, and feedback system, which was subsequently evaluated and refined through domain-expert input. Mapping the included sources to the taxonomy shows that current work is dominated by seizure detection and classification (prediction remains rare) and is largely situated in stationary, hospital ward settings, with comparatively limited evidence from home or residential contexts. Moreover, the field currently shows limited evaluation maturity: most systems remain proof of concept or pilot stage, while deployed solutions are rarely reported.

### Interpretation and Implications

Building on these principal findings, we derive a set of implications for research and practice (summarized in Multimedia Appendix 12), aimed at supporting researchers, clinicians, developers, policymakers, and patient advocacy groups.

#### Predictive Approaches Remain Underrepresented

Across the reviewed literature, vision-based seizure monitoring is predominantly framed as a recognition task focused on detection and classification, whereas prediction is rarely examined. This aligns with prior work describing seizure monitoring as primarily reactive rather than anticipatory (eg, time-to-event) in its current operationalization [3,5,17]. Potential explanations discussed in the literature include the limited specificity of preictal visual cues, constraints in obtaining sufficiently annotated preictal recordings, and difficulties in achieving robust generalization across patients and real-world settings [13]. Moreover, the most reliable visual markers typically appear at or after ictal onset, inherently biasing research toward detection rather than forecasting [5,51]. Addressing this gap requires methodological advances, multimodal data integration, and careful validation of predictive accuracy in real-world settings, which has been achieved outside vision-based monitoring systems [72].

#### Clinical and Patient-Centered Translation Remains Uneven

The evidence base emphasizes stationary clinical environments and professional workflows, while home or residential contexts are comparatively less represented. This pattern is consistent with a field in which technical feasibility and controlled evaluation conditions often precede broader implementation, particularly when systems must operate under variable lighting, camera placement, occlusion, and caregiver routines outside specialized clinical units [5,22]. Clinical readiness is a prerequisite for successful out-of-clinic use [22,67]. Establishing efficacy, safety, and operability in clinical workflows should come before extending to home environments, where variability and resource constraints otherwise amplify failure modes [5,22]. Sustained adoption in home environments hinges on usability and human factors, such as simple setup, low cognitive and technical demands, clear feedback, and an alarm burden that is manageable and fits with caregiver routines [3,16,22]. We therefore recommend co-design with patients and caregivers, treating out-of-clinic expansion as a distinct development step, requiring explicit attention to robustness, usability, and operational support.

#### Nonmotor Seizures and Multimodal Sensing Remain Less Often Addressed

Body-focused tracking and monitoring of the sleeping area are common targets, reflecting the clinical emphasis on nocturnal seizure detection. In contrast, more advanced or multimodal approaches, such as face tracking, room-wide overviews, or recognition of nonmotor phenomena, are less frequently described, indicating that visual monitoring remains narrow in scope and often captures overt motor activity in controlled settings. Current systems rely on visual cues to capture motor symptoms, with less attention to subtler phenomena that require physiological, audio, or multimodal data fusion [5,45,73]. Data fusion (eg, EEG and accelerometers) and adaptive filtering techniques can improve robustness [6,13]. Future research should expand beyond motor seizure detection and explore integrated sensing pipelines capable of handling diverse seizure presentations to increase noise resistance for real-world deployment.

#### System Maturity Highlights Gaps in Privacy and Regulatory Preparedness

Many solutions remain at the proof-of-concept stage, with limited progress toward certified medical devices. Privacy and security measures are also inconsistently reported: anonymization, pseudonymization, or encryption practices are seldom described as explicit system properties. In the European context, this is notable because data protection obligations under the General Data Protection Regulation and emerging requirements under the EU AI Act place increasing emphasis on governance, transparency, and safeguards for high-risk medical AI systems as they move toward deployment [74]. Work aiming for implementation may therefore benefit from integrating privacy-by-design and documentation-ready development practices earlier in the development.

#### Real-Time Feedback and Alerting Mechanisms Remain Uncommon

Many systems emphasize retrospective review and post hoc analysis, whereas real-time monitoring and responsive feedback mechanisms are less commonly addressed. This emphasis suggests that current solutions are often optimized for documentation and clinical review rather than time-critical response. For deployment-oriented use cases, future systems should specify the intended feedback objective (eg, documentation vs immediate escalation) and evaluate feedback as a system-level property, including latency, notification logic, and false alarm burden. Some existing systems already report latency and computational inefficiencies [5,45]. Optimizing real-time architectures through edge computing, efficient signal processing, and context-aware AI may reduce latency and support more reliable real-time operation in both clinical and home-based settings [62].

Taken together, these findings indicate a field that is technologically advanced in seizure recognition yet often focused on professional, stationary, and retrospective use cases. Important opportunities for predictive, patient-centered, multimodal, and privacy-preserving solutions remain largely unaddressed. These results suggest the need for standardized reporting of feedback latency, notification channels, escalation logic, and end-user targets to enable comparability and to assess clinical readiness. From a theoretical perspective, these patterns support the need for a maturity-oriented framework that integrates deployment setting, system maturity, data provenance, task type (detection, classification, and prediction), and reporting quality as core explanatory dimensions. The taxonomy developed in this scoping review operationalizes these dimensions and thereby contributes to conceptual standardization in the field. Overall, the implications outline a pathway from detection-focused development toward robust, patient-centered, explainable, and regulation-ready systems with the potential to advance epilepsy care, while preserving the empirical constraints and opportunities identified by the taxonomy.

### Limitations

This scoping review has several limitations. Despite a rigorous approach, the scoping review may still reflect selection bias because it was screened and selected by the reviewers, restricted to 3 bibliographic databases, and relied on predefined keywords and inclusion criteria. The exclusion of studies published in languages other than English has the potential to constitute an additional bias. Although the scoping review was conducted across 3 key databases commonly used in health and AI research, scoping reviews may benefit from an even more extensive search strategy, incorporating additional databases or gray literature, to further enhance methodological completeness. However, expert validation and iterative development ensure its robustness. Furthermore, the iterative E2C methodology inherently relies on interpretative refinements, introducing contextual dependencies. Nonetheless, this approach is well established in taxonomy development, allowing adaptability to technological and clinical advancements. The status quo on market-ready solutions appears limited in the landscape of AI monitoring systems that are based on visual data. NELLI [39] provided a basis for the iteration of practical assessment. To obtain a more comprehensive perspective on additional market solutions in the close field, the focus was directed toward a video-EEG solution known as SEER [24]. The performance metrics within AI models pose another challenge, given the multitude of metrics that can be considered. Furthermore, despite the underreporting of privacy and cryptographic safeguards in the literature, a jurisdiction-specific compliance analysis was not undertaken (eg, AI Act). This work does not include a detailed, jurisdiction-specific compliance mapping, which will be addressed in a dedicated follow-up study. Finally, the validation to date relied on the contributions of domain experts; patient and informal caregiver perspectives, and prospective real-world deployments were not yet included.

### Conclusions

This scoping review points to a broader transition problem in vision-based epilepsy monitoring: while algorithmic feasibility for seizure recognition is increasingly demonstrated, translation into deployable, trustworthy systems remains constrained by system-level requirements. Progress toward deployment in this domain will likely depend less on incremental model improvements and more on development and evaluation practices that explicitly address readiness for real-world operation, including robustness under uncontrolled conditions, workflow fit, feedback and escalation design, and governance mechanisms that make privacy protection and safety assurance auditable and maintainable over time.

The developed taxonomy provides a shared reference structure for describing and evaluating vision-based epilepsy monitoring technologies end to end. Its innovation lies in integrating application context with visual analysis, AI modeling, system architecture, and feedback design within a single classification framework. Where prior work has often centered on algorithms, datasets, or modality-specific performance summaries, this taxonomy enables consistent, system-level characterization across research prototypes and emerging solutions. By standardizing how systems are characterized across dimensions and characteristics, it can support benchmarking and implementation-focused evaluation (including procurement considerations) and guide implementation priorities. For implementation stakeholders, the taxonomy can serve as a structured checklist to align a system’s intended use and deployment setting with required architecture, feedback design, evaluation evidence, and safeguards, thereby informing selection, integration, and rollout planning. More broadly, the taxonomy supports progress in a diverse and fast-evolving field by making system boundaries, intended use, and evaluation targets explicit and comparable across studies. This can help shift the literature from model-centered reporting toward system-level evidence that can be interpreted across contexts, strengthening the basis for synthesis, replication, and translation. In this sense, the taxonomy is not only a classification tool but also a practical framework to support more consistent study design, reporting, and deployment-oriented evaluation in future research.

## Supplementary material

10.2196/83895 Multimedia Appendix 1 Exemplary schematic flow of monitoring systems based on visual data with artificial intelligence models.

10.2196/83895 Multimedia Appendix 2 Synthesis of the extended taxonomy design process: demonstration and evaluation aligned with 3 guiding questions of Design Science Research (table based on Nickerson et al [26]).

10.2196/83895 Multimedia Appendix 3 Overview of all iterations with the identified dimensions during the taxonomy development.

10.2196/83895 Multimedia Appendix 4 Search method and search strategies for all databases.

10.2196/83895 Multimedia Appendix 5 Comprehensive data extraction tables for taxonomy dimensions.

10.2196/83895 Multimedia Appendix 6 Comprehensive data extraction tables for study levels.

10.2196/83895 Multimedia Appendix 7 Coding template and detailed frequency analysis across all included studies.

10.2196/83895 Multimedia Appendix 8 Study sample selection of experts from practice and research.

10.2196/83895 Multimedia Appendix 9 Data charting of the study results.

10.2196/83895 Multimedia Appendix 10 Evidence maps of the study results.

10.2196/83895 Multimedia Appendix 11 Taxonomy application example for NELLI.

10.2196/83895 Multimedia Appendix 12 Main findings and implications for research and practice.

10.2196/83895 Checklist 1 PRISMA-S checklist.

## Abbreviations

AI: artificial intelligence
C2E: conceptual-to-empirical
CNN: convolutional neural network
DL: deep learning
E2C: empirical-to-conceptual
EEG: electroencephalogram
ETDP: extended taxonomy design process
GAIDeT: Generative Artificial Intelligence Delegation Taxonomy
Gen-AI: generative artificial intelligence
LSTM: long short-term memory
MeSH: Medical Subject Headings
PCC: population, concept, and context
PRESS: Peer Review of Electronic Search Strategies
PRISMA-ScR: Preferred Reporting Items for Systematic Reviews and Meta-Analyses extension for Scoping Reviews
RQ: research question
