# Supplementary figures and images for: Vision-Based Artificial Intelligence Technologies for Epilepsy Monitoring: Scoping Review and Taxonomy Development Study
Source: J Med Internet Res. 2026 Jun 24;28:e83895. doi: 10.2196/83895 (PMC13293478; doi:10.2196/83895)

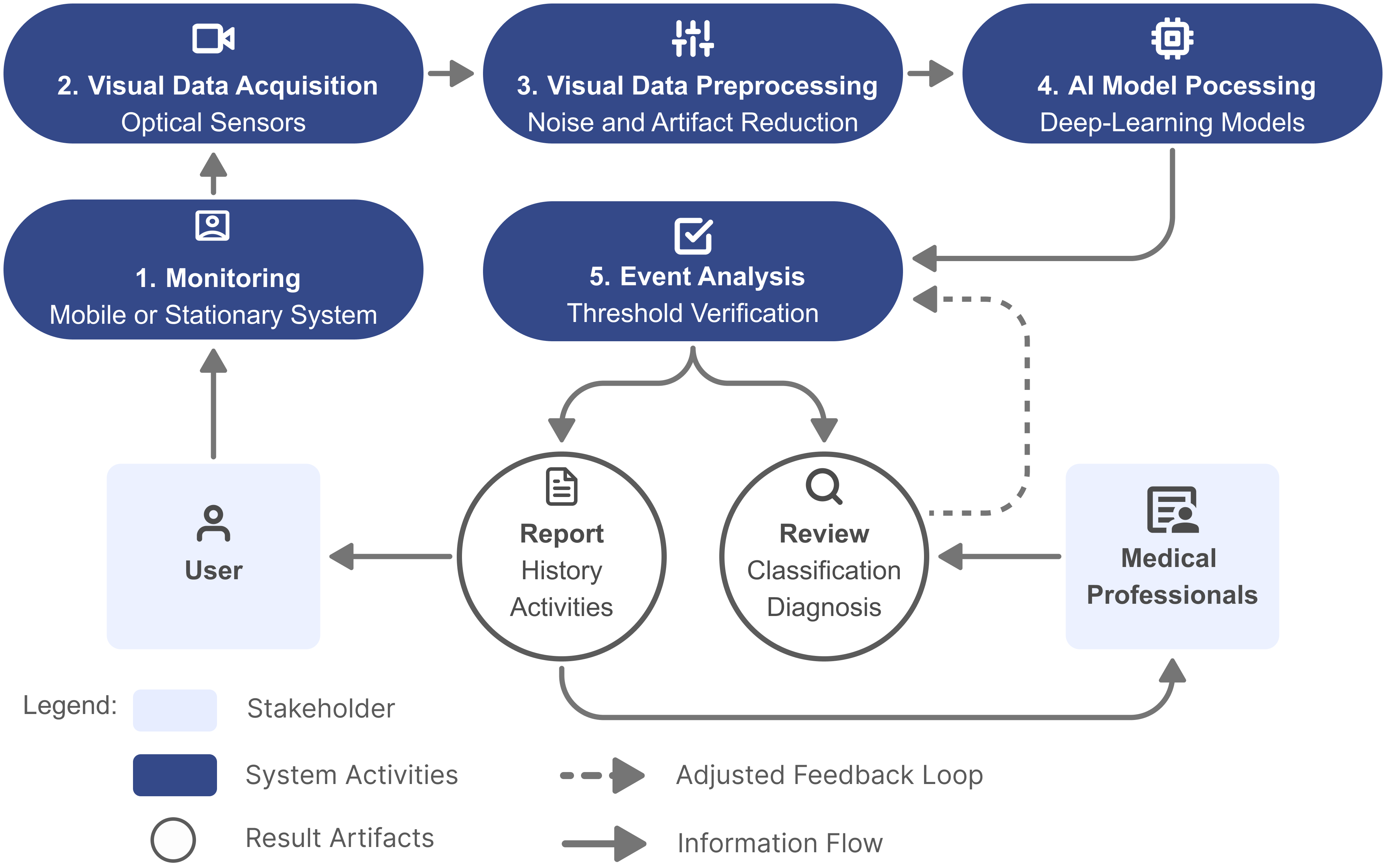

Supplement: Multimedia Appendix 1 [file jmir-v28-e83895-s001.png]

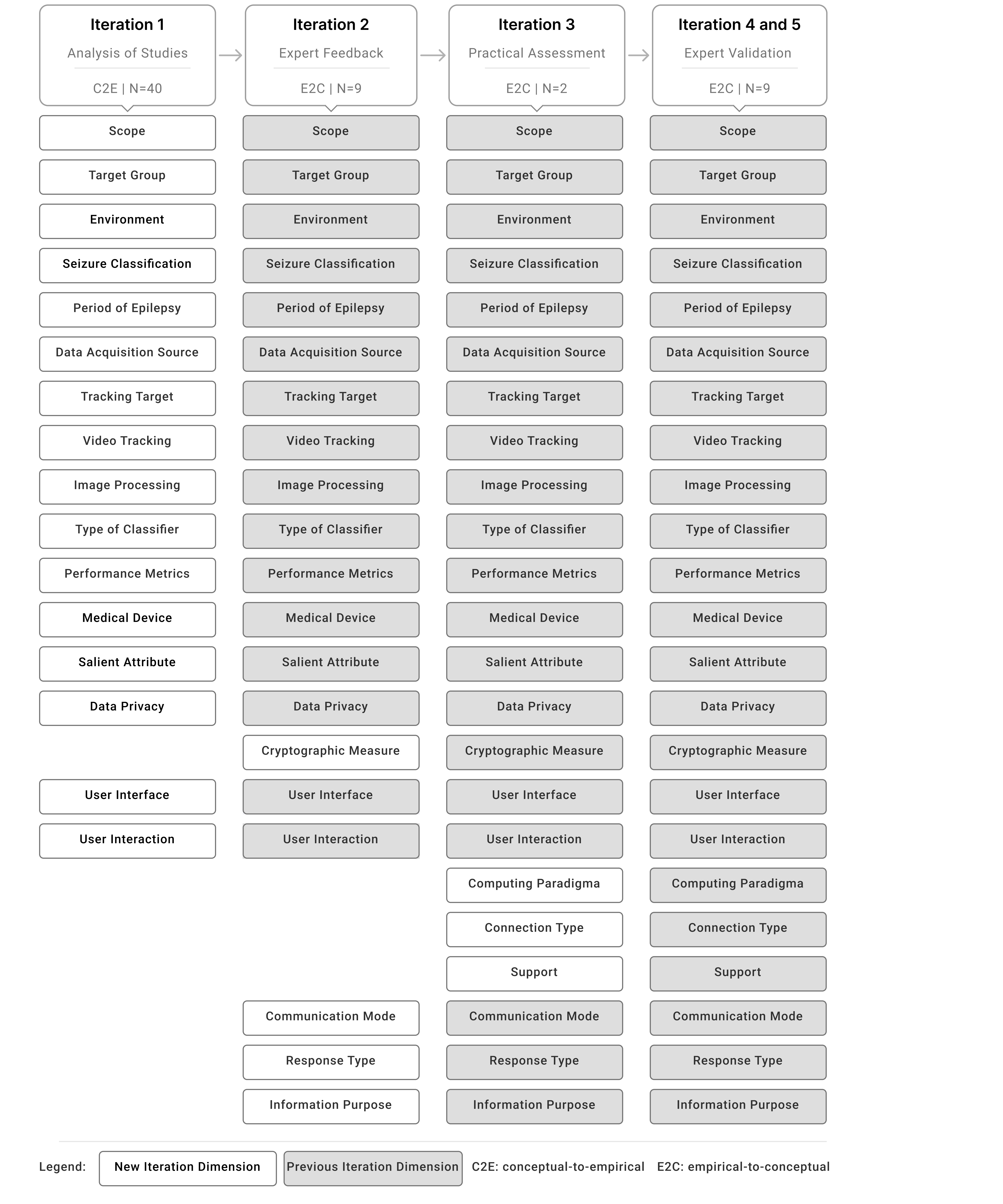

Supplement: Multimedia Appendix 3 [file jmir-v28-e83895-s003.png]
