# Supplementary material for: Vision-Based Artificial Intelligence Technologies for Epilepsy Monitoring: Scoping Review and Taxonomy Development Study
Source: J Med Internet Res. 2026 Jun 24;28:e83895. doi: 10.2196/83895 (PMC13293478; doi:10.2196/83895)
Supplement: Multimedia Appendix 2 [file jmir-v28-e83895-s002.pdf]

Synthesis of the ETDP: Demonstration and evaluation aligned with three guiding questions of Design Science Research (table based on Nickerson et al. [16]).

<table>
<tr><td rowspan="2"></td><td>Why</td><td colspan="3">How</td><td>What</td></tr>
<tr><td>concerning function</td><td>concerning environment</td><td>concerning timing</td><td>concerning method</td><td>concerning criteria</td></tr>
<tr><td rowspan="2">Check objective ending conditions</td><td>Formative</td><td>Artificial and naturalistic</td><td>Ex ante</td><td>Consensus among the taxonomy designers, Delphi-study and practical assessment</td><td>(1) all identified objects within the taxonomy must have been examined; (2) each characteristic in every dimension must classify at least one object; (3) no additional dimensions or characteristics can be introduced in the final iteration and (4) dimensions, characteristics and cell combinations must be unique and non-redundant.[15]</td></tr>
<tr><td colspan="5">Is it a taxonomy? In the course of developing a taxonomy, researchers must provide an objective demonstration that the current version of the taxonomy satisfies the necessary conditions for qualifying as a taxonomy.</td></tr>
<tr><td rowspan="2">Check subjective ending conditions</td><td>Formative</td><td>Artificial</td><td>Ex ante</td><td>Delphi-study</td><td>Taxonomy must be concise, comprehensive, robust, explainable and extensible (Nickerson et al., 2013)</td></tr>
<tr><td colspan="5">Is the taxonomy applicable? During the process of taxonomy construction, researchers critically examine, guided by their expert judgment, whether the existing version of the taxonomy fulfils the conditions required to qualify as an applicable taxonomy.</td></tr>
<tr><td rowspan="2">Configure evaluation</td><td>Summative</td><td>Naturalistic</td><td>Ex post</td><td>Delphi-Study</td><td>Usefulness of taxonomy for target group</td></tr>
<tr><td colspan="5">Is the taxonomy useful? Following the construction of a taxonomy, its utility is assessed by examining whether the final version satisfies the sufficiency condition and established evaluation criteria. This evaluation is typically informed by feedback from potential users to determine whether the taxonomy can be considered useful in practice.</td></tr>
</table>
