# Supplementary material for: Vision-Based Artificial Intelligence Technologies for Epilepsy Monitoring: Scoping Review and Taxonomy Development Study
Source: J Med Internet Res. 2026 Jun 24;28:e83895. doi: 10.2196/83895 (PMC13293478; doi:10.2196/83895)
Supplement: Multimedia Appendix 4 [file jmir-v28-e83895-s004.pdf]

Search method and search strategies for all databases.

**Database**	**Search String**
Scopus	TITLE-ABS-KEY (("epilep*" OR "seizure*" OR "epileptic seizure*" OR "seizure disorder*" OR "tonic-clonic" OR "tonic clonic" OR "ictal" OR "convulsion*" OR "generalized tonic-clonic" OR "grand mal") AND ("AI" OR "artificial intelligence" OR "machine learning" OR "deep learning" OR "deep neural network*" OR "convolutional neural network*" OR "CNN") AND ("predict*" OR "monitor*" OR "forecast*" OR "observ*" OR "recognit*" OR "alert*" OR "track") AND ("video*" OR "camera*" OR "depth sensor*" OR "3D camera*" OR "vision-based" OR "vision based" OR "visual*" OR "motion*" OR "computer vision" OR "pose estimation")) AND PUBYEAR > 2012
PubMed	(((epilep*[tiab] OR seizure*[tiab] OR "epileptic seizure*"[tiab] OR "seizure disorder*"[tiab] OR "tonic-clonic"[tiab] OR "tonic clonic"[tiab] OR ictal[tiab] OR convulsion*[tiab] OR "generalized tonic-clonic"[tiab] OR "grand mal"[tiab])<br>AND<br>(AI[tiab] OR "artificial intelligence"[tiab] OR "machine learning"[tiab] OR "deep learning"[tiab] OR "deep neural network*"[tiab] OR "convolutional neural network*"[tiab] OR CNN[tiab])<br>AND<br>(predict*[tiab] OR monitor*[tiab] OR forecast*[tiab] OR observ*[tiab] OR recognit*[tiab] OR alert*[tiab] OR track[tiab])<br>AND<br>(video*[tiab] OR camera*[tiab] OR "depth sensor*"[tiab] OR "3D camera*"[tiab] OR "vision-based"[tiab] OR "vision based"[tiab] OR visual*[tiab] OR motion*[tiab] OR "computer vision"[tiab] OR "pose estimation"[tiab]))<br>OR<br>(("Epilepsy"[MeSH Terms] OR<br>"Seizures"[MeSH Terms] OR<br>"Epilepsy, Tonic-Clonic"[MeSH Terms] OR<br>"Status Epilepticus"[MeSH Terms])<br>AND<br>("Artificial Intelligence"[MeSH Terms] OR<br>"Machine Learning"[MeSH Terms] OR<br>"Deep Learning"[MeSH Terms] OR<br>"Neural Networks, Computer"[MeSH Terms] OR<br>"Pattern Recognition, Automated"[MeSH Terms] OR<br>"Diagnosis, Computer-Assisted"[MeSH Terms])<br>AND<br>("Video Recording"[MeSH Terms] OR<br>"Motion Capture"[MeSH Terms] OR<br>"Time-Lapse Imaging"[MeSH Terms] OR<br>"Image Processing, Computer-Assisted"[MeSH Terms] OR<br>"Image Interpretation, Computer-Assisted"[MeSH Terms])<br>AND<br>("Monitoring, Physiologic"[MeSH Terms] OR<br>"Neurophysiological Monitoring"[MeSH Terms] OR<br>"Remote Patient Monitoring"[MeSH Terms] OR<br>"Forecasting"[MeSH Terms])))<br>AND ("2013/01/01"[dp] : "3000/12/31"[dp])

Web of Science	(((ALL=(("epilep*" OR "seizure*" OR "epileptic seizure*" OR "seizure disorder*" OR "tonic-clonic" OR "tonic clonic" OR "ictal" OR "convulsion*" OR "generalized tonic-clonic" OR "grand mal"))) AND ALL=(("AI" OR "artificial intelligence" OR "machine learning" OR "deep learning" OR "deep neural network*" OR "convolutional neural network*" OR "CNN"))) AND ALL=(("predict*" OR "monitor*" OR "forecast*" OR "observ*" OR "recognit*" OR "alert*" OR "track"))) AND ALL=(("video*" OR "camera*" OR "depth sensor*" OR "3D camera*" OR "vision-based" OR "vision based" OR "visual*" OR "motion*" OR "computer vision" OR "pose estimation"))

In addition to the exclusion criteria, the following **inclusion criteria** were applied during the systematic literature review:

1. Machine Learning Studies: Publications focusing on machine learning, particularly those comparing or applying artificial intelligence (AI) techniques, were included even if the system description was limited.
2. Audiovisual Content: Studies involving audiovisual data that enhance epilepsy monitoring through video were included.
3. Video-EEG Studies: Studies combining video with electroencephalography (video-EEG) were included, as this approach is widely used in clinical practice for comprehensive seizure assessment.
4. Patient Population: Studies involving human participants diagnosed with epilepsy were included.
5. Publication Date: Only studies published after 2012 were included, corresponding with the emergence of deep learning methodologies.
6. System Descriptions and Proof-of-Concept Studies: Papers presenting proof-of-concept systems or detailed system descriptions were included to capture both conceptual and applied developments in AI-based epilepsy monitoring.

The following **exclusion criteria** were applied during the systematic literature review:

1. Language: Studies published in languages other than English were excluded.
2. Non-Video Monitoring: Studies that did not utilize video monitoring or lacked any audiovisual component were excluded.
3. Non-AI Studies: Studies that did not incorporate artificial intelligence or machine learning methods were excluded.
4. Wearable-Only Systems: Research focusing solely on wearable devices (e.g., smartwatches) was excluded, unless combined with video or camera systems (e.g., SEER).
5. Publication Date: Studies published before 2012 were excluded.
6. Reviews were excluded.

The following **limits** were applied during the systematic literature review:

1. Document type: article
2. Language: English

Search History:

**Version**	**Run date**	**Database**	**Strategy summary**	**Limits/filters applied**
V1	06 Jan 2025	Scopus; PubMed; Web of Science Core Collection	Search string: (“epilepsy”) AND (AI/ML/DL) AND (prediction/monitoring/forecasting/observation) AND (video/visual/motion)<br><br>developed de novo; not adapted from prior reviews	English; document type=article; year >2012
V2	01 Oct 2025	Scopus; PubMed; Web of Science Core Collection	Same core string as V1; reported as “redone” during revision	English; document type=article; year >2012
V3	17 Nov 2025	Scopus; PubMed (plus MeSH); Web of Science Core Collection	Expanded string with epilepsy synonyms + AI/DL terms + task terms + video/vision terms (incl. pose estimation); PubMed additionally complemented with MeSH	English; document type=article; year >2012
V4 (final; evidence base)	16 Jan 2026	Scopus; PubMed (plus MeSH); Web of Science Core Collection	Same expanded concept blocks as V3	English; document type=article; year >2012
