# Supplementary material for: Vision-Based Artificial Intelligence Technologies for Epilepsy Monitoring: Scoping Review and Taxonomy Development Study
Source: J Med Internet Res. 2026 Jun 24;28:e83895. doi: 10.2196/83895 (PMC13293478; doi:10.2196/83895)
Supplement: Multimedia Appendix 5 [file jmir-v28-e83895-s005.pdf]

Column name	What to enter	codes
Scope	Intended functional purpose of the system	Detection; Classification; Prediction; Not reported
Target_Group	Primary intended users / stakeholders	Epilepsy patients; Caregivers; Medical professionals; Data scientists; Not reported
Environment	Intended deployment environment / mobility	Stationary; Mobile; Not reported
Seizure_Classification	Seizure symptom type(s) targeted by the system	Non-motor symptoms (sensory, emotional, autonomic, absences); Motor symptoms (focal motor, e.g. clonic, tonic or generalized motor: tonic-clonic seizures); Not reported
Period_of_Epilepsy	Epilepsy/seizure phase addressed	Inter-ictal; Pre-ictal; Ictal; Post-ictal; Not reported
Data_Acquisition_Source	Sensor / modality used to acquire input data	Depth sensors; Infrared; 2D; 3D; Video-EEG; EKG; Audio; Not reported
Tracking_Target	What is tracked in the video scene	Face; Body; Sleeping area; Room overview; Not reported
Video_Tracking	Video tracking approach / target representation	Movement dynamics; Biomechanics characteristics (e.g. speed/acceleration patterns, movement amplitude); Movements of interest (MOI); Region of interest (ROI); Head movement detection (HMD-Modell); Simple keypoint system (SKPS); Appearance and feature based methods; Not reported
Image_Processing	Low-/mid-level image processing approach	Frame Differencing; Optical flow; Spatiotemporal Interest Points; Contrast Based Analysis; Not reported
Type_of_Classifier	Machine learning / AI model used for classification	Support vector machine; Multilayer perceptron; Convolutional neural network; Random forest; Gaussian mixture model; Long short-term memory (LSTM) classifier; I3D classifier; Other type of classifier; Not reported
Performance_Metrics	Reported performance indicators	Accuracy; Sensitivity; Specificity; Precision; Recall; False positive rate (= false alarm rate); Area Under Curve; F1-Score (metric combines precision and recall to detect performance overall); Not reported
Medical_Device_Status	Market/regulatory maturity label	Certified; Proof of concept; Not certified; Not reported
Salient_Attributes	Notable product/system characteristics emphasized	Environmental robustness; Cost-efficiency: low costs (no long hospital stays needed); Real time analysis; Ease of use / Usability; High system performance; Not reported

Data_Privacy	Stated privacy approach / data protection measures	Pseudonymization; Anonymization; No privacy preserving measures; Synthetic data; Not reported
Cryptographic_Measure	Encryption/security measures stated	Encryption in transit; Encryption at rest; No encryption; Not reported
User_Interface	Interface/channel provided to users	Voice Assistant; Web platform; Mobile applications; Wearable device; Desktop Application; Not reported
User_Interaction	Interaction style between system and user	Reporting; Adaptive; No interaction; Interactive; Not reported
Computing_Paradigm	Where computation is performed	Cloud-based platforms; Edge-based platform; Local on device; Not reported
Connection_Type	Connectivity technology used	Wi-Fi; Built-in-Modem; Ethernet; Bluetooth; Not reported
Support	Support and service mechanisms offered	On-Call; System Setup; Help Centre; Daily Technical Checks; Expert Data Review; Chat; Not reported
Communication_Mode	How/when the system communicates outputs	Real-Time; Periodic; Event-Based; On-Demand; Not reported
Response_Type	Output modality of feedback	Visual; Auditory; Haptic; Text-Based; Not reported
Information_Purpose	Intended purpose of the provided information	Alerting / Warning; Performance Evaluation; Recommendation; User Learning; Not reported
