# Supplementary material for: Vision-Based Artificial Intelligence Technologies for Epilepsy Monitoring: Scoping Review and Taxonomy Development Study
Source: J Med Internet Res. 2026 Jun 24;28:e83895. doi: 10.2196/83895 (PMC13293478; doi:10.2196/83895)
Supplement: Multimedia Appendix 6 [file jmir-v28-e83895-s006.pdf]

Column name	What to enter	codes
Year	Publication year	YYYY
Setting	Where monitoring is intended/used	Home; Hospital ward; residential setting; Lab/Simulated; Not
Study_Design	Overall design type	Method development; Retrospective; Prospective observational; Feasibility/Pilot; RCT/Controlled; Implementation study; Not reported
Evaluation_Maturity	How close to real-world use	Concept only; Lab prototype; Pilot in real setting; Deployed/Operational; Not reported
Data_Source_Type	Provenance of data	Prospective collected; Retrospective clinical; Public dataset; Staged/Scripted; Mixed; Not reported
Sample_Type	Who was monitored	Patients; volunteers; Mixed; None/Not applicable; Not reported
Sample_Size	N (or other unit)	Numeric N; or “X videos / X hours”; or Not reported
