# Supplementary material for: Vision-Based Artificial Intelligence Technologies for Epilepsy Monitoring: Scoping Review and Taxonomy Development Study
Source: J Med Internet Res. 2026 Jun 24;28:e83895. doi: 10.2196/83895 (PMC13293478; doi:10.2196/83895)
Supplement: Multimedia Appendix 8 [file jmir-v28-e83895-s008.pdf]

Study sample selection of experts from practice and research (own table).

Practitioners	Role and Expertise	Professional Experience	Age	Sex
P1	AI-Solutions-Architect	9 years	33	f
P2	AI Engineer & TechLead	10 years	40	m
P3	Specialist in neurology and epileptology	24 years	52	m
Researchers	Research Scope and Expertise	Professional Experience	Age	Sex
R1	Research Associate / Method expert	5 years	34	m
R2	Research Associate / AI & eHealth	5 years	30	f
R3	Research Associate / Data security	10 years	33	f
R4	Research Associate / AI & eHealth	2 years	27	f
R5	Research Associate / Method expert	4 years	31	f
R6	Research Associate / AI & eHealth	7 years	32	f
