# Supplementary material for: Vision-Based Artificial Intelligence Technologies for Epilepsy Monitoring: Scoping Review and Taxonomy Development Study
Source: J Med Internet Res. 2026 Jun 24;28:e83895. doi: 10.2196/83895 (PMC13293478; doi:10.2196/83895)
Supplement: Multimedia Appendix 10 [file jmir-v28-e83895-s010.pdf]

Evidence maps of the study results.

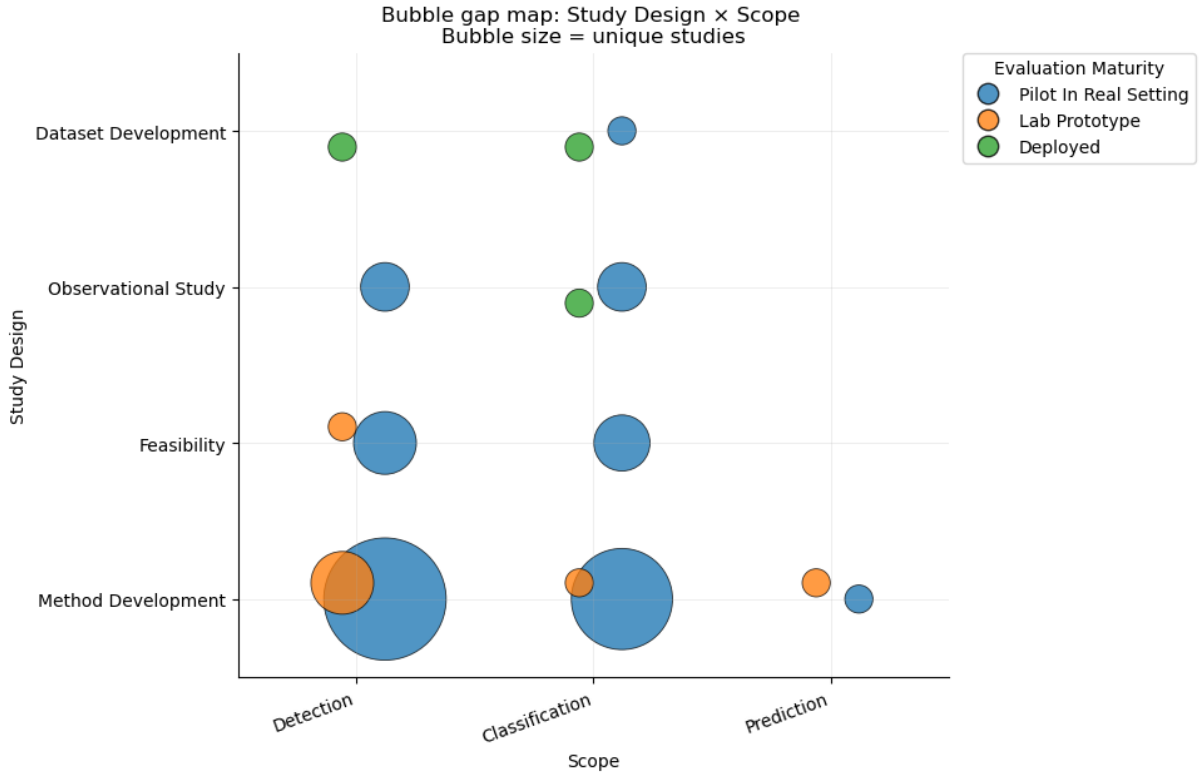

Evidence map of included studies by study design and functional scope; bubble size indicates number of studies and color indicates evaluation maturity.

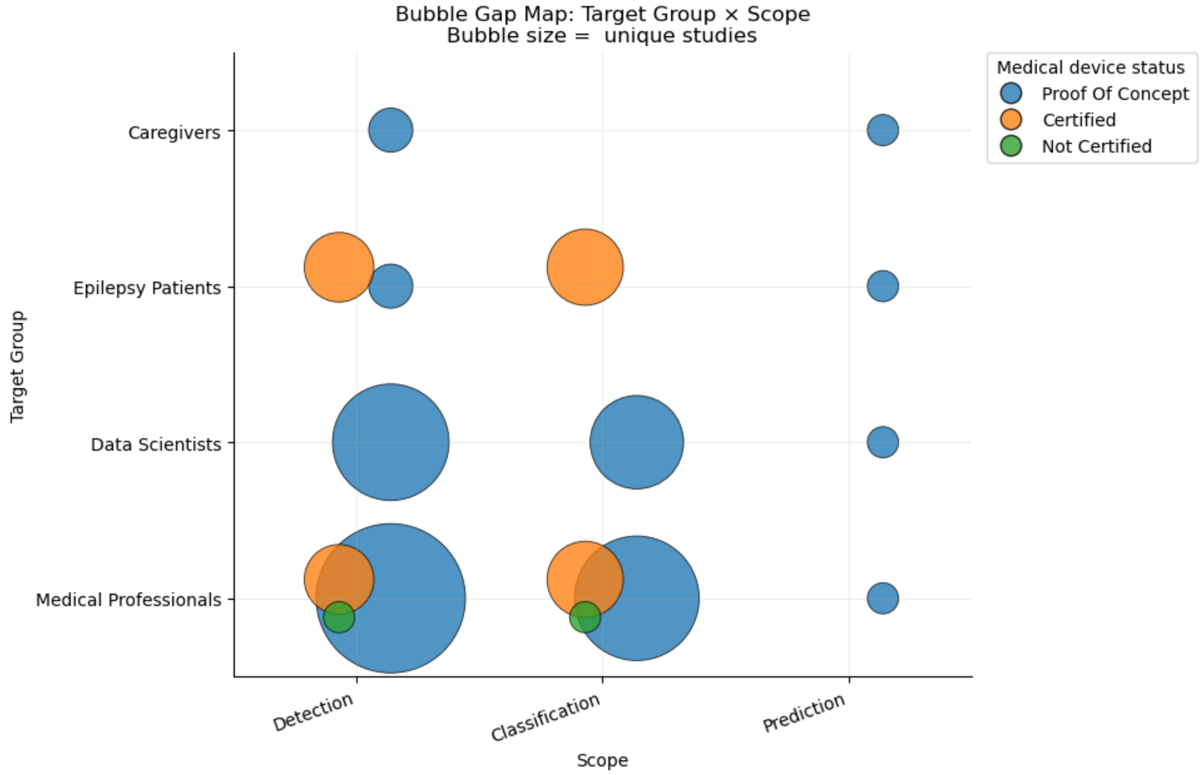

Evidence map of included studies by target group and functional scope; bubble size indicates number of studies and color indicates medical device status.

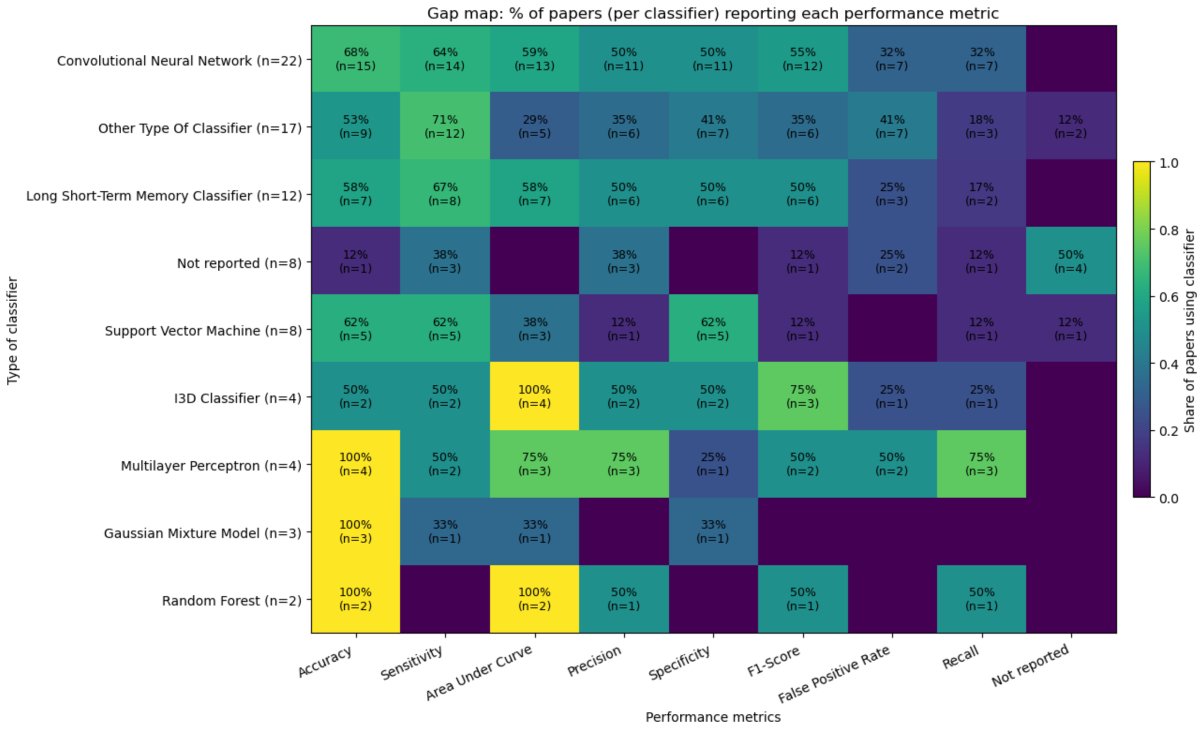

Gap map of performance reporting by classifier type; cells show the proportion (and count) of studies reporting each performance metric.
