# Supplementary material for: Vision-Based Artificial Intelligence Technologies for Epilepsy Monitoring: Scoping Review and Taxonomy Development Study
Source: J Med Internet Res. 2026 Jun 24;28:e83895. doi: 10.2196/83895 (PMC13293478; doi:10.2196/83895)
Supplement: Multimedia Appendix 11 [file jmir-v28-e83895-s011.pdf]

Taxonomy application example for NELLI.

Aspect	Dimension	Characteristics							
Application and Context	Scope	Detection	Classification	Prediction					
	Target Group	Epilepsy Patients	Caregivers	Medical Professionals	Data Scientists				
	Environment	Stationary	Mobile						
	Seizure Classification	Non-Motor Symptoms	Motor Symptoms						
	Period of Epilepsy	Inter-ictal	Pre-ictal	Ictal	Post-ictal				
	Data Acquisition Source	Depth-Sensors	Infrared	2D Camera	3D Camera	Video-EEG	ECG	Audio	
Visual Analysis	Tracking Target	Body	Face	Sleeping Area	Room Overview				
	Video Tracking	MD	BC	MOI	ROI	HMD	SKS	Appearance and feature based methods	
	Image Processing	Optical Flow	Frame Differencing	Spatiotemporal Interest Points	Contrast Based Analysis				
AI Model	Type of Classifier	SVM	RF	MP	CNN	GMM	LSTM	I3D	Other Type of Classifier
	Performance Metrics	Accuracy	Sensitivity	Specificity	F1-Score	Precision	Recall	False positive rate	Area Under Curve
Market Identity	Medical Device	Certified	Not Certified	Proof of Concept					
	Salient Attribute	Environmental Robustness	Cost-Efficiency	Real-Time Analysis	Ease of Use	High System Performance			
	Data Privacy	Anonymization	Pseudonymization	No Privacy Preserving Measures	Synthetic Data				
	Cryptographic Measure	Encryption in Transit	Encryption at Rest	No Encryption					
System Architecture	User Interface	Web Platform	Voice Assistant	Mobile Application	Desktop Application	Wearable Device			
	User Interaction	Reporting	Interactive	Adaptive	No Interaction				
	Computing Paradigm	Cloud-based Platform	Edge-based Platform	Local on Device					
	Connection Type	Wi-Fi	Built-in-Modem	Ethernet	Bluetooth				
	Support	On-Call	System Setup	Help Center	Daily Technical Checks	Expert Data Review	Chat		
Feedback System	Communication Mode	Real-Time	Periodic	Event-Based	On-Demand				
	Response Type	Visual	Auditory	Haptic	Text-Based				
	Information Purpose	Alerting / Warning	Performance Evaluation	Recommendation	User Learning				

Legend:
EEG: Electroencephalogram; ECG: Electrocardiogram; MD: movement dynamics; BC: biomechanical characteristics; MOI: movement of interest; ROI: region of interest; HMD: head movement detection, SKS: simple keypoint system; SVM: support vector machine; RF: random forest, MP: multilayer perceptron, CNN: convolutional neural network, GMM: Gaussian mixture model, LSTM: long short-term memory; I3D: inflated 3D.
