# Supplementary material for: Vision-Based Artificial Intelligence Technologies for Epilepsy Monitoring: Scoping Review and Taxonomy Development Study
Source: J Med Internet Res. 2026 Jun 24;28:e83895. doi: 10.2196/83895 (PMC13293478; doi:10.2196/83895)
Supplement: Multimedia Appendix 12 [file jmir-v28-e83895-s012.pdf]

Main Findings, implications for research and practice (own table).

Main findings (MF)	*Implications for research*	*Implications for practice*
MF1: Vision-based seizure monitoring is largely framed as detection/classification, while prediction is rarely addressed.	IR1: Advance predictive, time-to-event modeling and multimodal datasets with robust real-world validation.	IP1: Develop early-warning approaches that enable anticipatory support while managing false alarms and uncertainty.
MF2: Evidence emphasizes stationary clinical settings and professional workflows; home/residential contexts are less represented.	IR2: Evaluate deployment in home/ambulatory settings and study human factors, usability, and caregiver routines via prospective studies.	IP2: Treat out-of-clinic deployment as a distinct step, requiring robustness, usability, and operational support.
MF3: Non-motor seizures and multimodal sensing are less often addressed; visual monitoring mainly captures overt motor activity.	IR3: Expand to diverse seizure presentations and multimodal sensing/data fusion, including noise-resistant algorithms.	IP3: Broader seizure coverage and multimodal robustness may improve diagnostic completeness and reduce missed events.
MF4: System maturity highlights gaps in privacy/security reporting and regulatory preparedness; many systems remain proof-of-concept.	IR4: Integrate privacy-by-design and documentation-ready development; study pathways from prototypes to certified tools.	IP4: Make privacy/security safeguards explicit system properties and align development with governance and regulatory expectations.
MF5: Real-time monitoring, feedback, and alerting are uncommon; systems often focus on retrospective review.	IR5: Design and benchmark real-time architectures, including latency, escalation logic, and alarm burden, supported by efficient computing.	IP5: Specify feedback objectives and evaluate system-level performance (e.g., latency and alarm burden) for deployment-oriented use.
